# Improvement of displacement estimation of breast tissue in ultrasound elastography using the monogenic signal

**DOI:** 10.1186/s12938-017-0313-3

**Published:** 2017-01-17

**Authors:** Taher Slimi, Ines Marzouk Moussa, Tarek Kraiem, Halima Mahjoubi

**Affiliations:** 1 Laboratory of Biophysics and Medical Technologies, High Institute of Medical Technologies of Tunis, University of Tunis El Manar, 9th Dr. Zouhair Essafi Street, 1006 Tunis, Tunisia; 2 Department of Medical Imaging and Radiology, University Hospital Center of Monji Slim, 2046 Marsa, Tunisia; 3 Department of Biophysics, Faculty of Medicine of Tunis, University of Tunis El Manar, 1007 Rabta, Tunisia; 4 Department of National Radiation Protection Center, Bab Sadoun Children’s Hospital, 1006 Tunis, Tunisia

**Keywords:** Breast static elastography, Displacement estimation, Monogenic signal, Shrinkage wavelets

## Abstract

**Background:**

In breast ultrasound elastography, tissues displacements estimation is obtained through a technique that follows the evolution of tissues under stress. However, during the acquisition of B-mode images, tissue displacements are often contaminated with multiplicative noise caused by changes in the speckle pattern in the tissue. Thus, the application of monogenic signal technique on the B-mode image in order to estimate displacement tissue, result in a presence of amplified noise in the deformation tissue image, which severely obscures the useful information. In this paper, we propose a new method based on the monogenic features, that is to improve the old monogenic signal (OMS) technique by improving the filtering step, so that the use of an effective denoising technique is enough to ensure a good estimation of displacement tissue. Our proposed method is based on the use of a robust filtering technique combined with the monogenic model.

**Methods:**

Two models of phantom elasticity are used in our test validation sold by CIRS company. In-vivo testing was also performed on the sets of clinical B-mode images to 20 patients including malignant breast tumors. Shrinkage wavelets has been used to eliminate the noise according to the threshold, then a guided filter is introduced to completely filter the image, the monogenic model is used after excerpting the image feature and estimating analytically the displacement tissue.

**Results:**

Accurate and excellent displacement estimation for breast tissue was observed in proposed method results. By adapting our proposed approach to breast B-mode images, we have shown that it demonstrated a higher performance for displacement estimation; it gives better values in term of standard deviation, higher contrast to noise ratio, greater peak signal-to-noise ratio, excellent structural similarity and much faster speed than OMS and B-spline techniques. The results of the proposed model are encouraging, allowing quick and reliable estimations.

**Conclusion:**

Although the proposed approach is used in ultrasound domains, it has never been used in the estimation of the breast tissue displacement. In this context, our proposed approach could be a powerful diagnostic tool to be used in breast displacement estimation in ultrasound elastography.

## Background

In recent years, ultrasound elastography has developed to characterize the viscoelastic properties of soft tissues. This imaging method is particularly promising to characterize pathologies such as carcinomas in the breast, that present a greater elasticity than the surrounding tissues [1].

Ultrasound elastography has a purpose to offer the tools allowing to doctors to make the best decisions suited to the pathologies in terms of diagnosis, detection or therapy. This technique makes it possible to quantify the mechanical properties of soft tissues by ultrasonic medical imaging [2]. Hence its objective is to correlate the mechanical properties of soft tissue with their healthy or pathological characteristics. Historically, this technique has existed for a very long time in a qualitative way. In fact, by palpation, doctors evaluate the hardness of the tissues and therefore evaluate its mechanical properties, like the elasticity. In this context the so-called static elastography was developed: imaging the internal deformation of the tissues under stress by ultrasound imaging [3].

In this axis, static ultrasound elastography is a very important technique that has proved its effectiveness in the medical environment; Focusing on ultrasound breast elastography, it was reported that the different breast tissues had different elastic characteristics of the glandular and connective tissue, and that it was possible to differentiate the intra-malignant or infiltrating malignant component from one malignant lesion using ultrasound elastography. However this technique suffers from noise and artifacts in the imaging system environment. The noise presents in the tissues displacements image degrades its quality and modifies the important details, related to texture and tissue morphology, which complicates the diagnosis in static ultrasound elastography [4].

From clinical application perspective, the elimination of speckle noise becomes a crucial step before proceeding with the medical diagnosis. The researchers are inspired to devote their efforts on this subject, in order to develop a technique to reduce the risk of misdiagnosis [5].

In the literature the researchers have developed several methods of estimating tissue displacements. The main method to estimate the displacement on B-mode images is block matching using cost functions. The pairing of blocks has a major disadvantage: It requires interpolation for sub-pixel estimation, so the time calculation is longer compared to the other displacement techniques [6]. Recently new techniques based on phase difference have been developed to estimate tissue displacements; however the results are always degraded by speckle noise [7].

To solve this problem, several filters have been developed in the literature in order to reduce the noise and the artifacts present in tissue displacements images, such as Frost filter, non-log-transformed generalized likelihood ratio method, Lee filter, Kuan filter, method and speckle reducing anisotropic diffusion [8]. But always, there are losses of partial content of image.

The application of local adaptive filters modifies slightly the information in the image and provides an inadequate filtering. Anisotropic diffusion filters have a high noise suppression capability; however, can create a strong smoothing of the tissue displacement image. The average nonlocal filters have a better filtering effect against speckle noise, but the algorithm complexity are generally very high, therefore they cannot meet the requirements of elastography [9].

Another solution using a multi-scale filtering approaches that they provide a good results, however they cannot make the optimum balance between speckle noise removal and preservation characteristics.

Finally, a technique of tissue displacements that we called it in this paper: OMS, which includes the DoP filter, this filter has reduced the noise in the image; however the noise is still present in the low frequency component and slightly deteriorates the result [10, 11].

In this approach, it is desirable to develop a technique that improves the performance of estimating tissue displacements with a good noise attenuation and better edge preservation as well as fast and reliable.

In this paper, we proposed a new approach which improves the OMS method. The proposed new approach exploits the shrinkage wavelets with a guided filter coupled combined with monogenic features. This proposed strategy improves the OMS method used in breast tissue displacement.

The results of in vivo and in vitro studies are presented to assess the proposed method. We show that the proposed model improved the OMS technique, and improved the diagnosis of breast pathology.

The performances of our proposed method are compared with OMS and BS methods; we show that our proposed method is more accurate, it gives better values in term of SD, higher CNR, greater PSNR, excellent SSIM and much faster speed than other methods.

The paper proceeds as follows: the adapted filtering technique and the monogenic signal theory of displacement estimation are resumed and presented in “Methods” section.

“Results” section shows the results on soft biological phantom designed for elastography and In-vivo breast images, we address also the comparison between the results obtained with our proposed method and those obtained with OMS and BS methods.

“Discussion” section shows the discussion of results.

Concluding remarks are left to “Conclusion” section.

## Methods

### Displacement estimation enhancement based on monogenic signal method

#### Image filtering

When a tissue is mechanically subjected to a quasi-static compression, the internal stresses are defined by the boundary conditions and by the intrinsic properties of the tissue [12].

The displacements generated by compression can be evaluated by ultrasonic wave when the scanned area is diffusing [13].

It is an area in which an ultrasonic pulse encounters on its way impedance inhomogeneities could create a return pulse. The complex interference of these reflected waves forms called “acoustic speckle”. The speckle is a particular noise that is found in all ultrasonic images, it is a multiplicative noise, that the echo summations are independent of the coefficient of background reflectivity; it depend only on the micro-relief. In this context, many authors have made contributions to improve speckle filtering techniques on ultrasonic data [7]. The crucial goal in any filtering is to remove the noise without losing the resolution of the image. In this framework, filtering of B-mode images is a very essential step before going to estimate the displacement of breast tissue. We may apply the shrinkage wavelets with a guided filter, in order to suppress the speckle noise and protect the details of the image against the degradation; our strategy will necessarily improve the displacement estimation of tissue in static ultrasound elastography [14].

##### Threshold selection

It is shown that it is possible to perform a wavelet decomposition of an image, and then reconstruct the image from its wavelet coefficients [15]. The wavelet coefficients mark the discontinuities that occur in the image, therefore they correspond to image details.

If the selected threshold value is below the noise wavelet coefficients, then the final image will still contain noise. Otherwise, if the threshold value is wider than the wavelet coefficients of certain details, the result is an image that has lost significant details for clinical diagnosis [16], so the threshold has a direct effect on the quality of the final image.

In order to achieve a compromise between noise suppression and preservation of image details, there is shown in the literature that the researchers improved the classic wavelet threshold function; the new version of this classic expression has proved effective against noise in medical ultrasound images [17].

In this paper, we propose the use of this improved function as shown below;

1 $${\text{T}}_{\text{j}} = {\text{a}}_{\text{j}} \upsigma_{\text{n}} \sqrt {2\log {\text{M}}}$$

where T is a threshold, j(1,2….,J) are the decomposition layers of wavelet transformation, J denotes the largest decomposition layers, a_j_ represents the adaptive parameter of the j layer, and is determined experimentally, σ_n_ denotes the standard deviation of noise in wavelet domain, and M is the number of the wavelet coefficients in the corresponding wavelet domain.

##### Wavelet shrinkage technique

The wavelet threshold shrinkage algorithm is applied here using Bayesian maximum a posterior estimation, there is shown in the literature that this improved expression of wavelet threshold shrinkage gives better results than Soft and Hard threshold [18], the reason why, we decided to use this technique to improve the final results of breast B-mode ultrasound images.

2 $$\hat{g} = \left\{ \begin{array}{ll} 0 &\quad {\text{f}} \le {\text{T}}_{\text{j}} \\ {{\text{sign}}\left( {\text{f}} \right) \cdot { \hbox{max} }\left( {\left| {\text{f}} \right| - \frac{{\sqrt {\sigma_{\text{n}}^{2} + 2\sigma_{\text{n}}^{2} \sigma_{\text{g}}^{2} } }}{{\sqrt {2\sigma_{\text{g}} } }},0} \right)} &\;\;\;\;{\text{f}} >{\text{T}}_{\text{j}} \\ \end{array} \right.$$

where ${\hat{\text{g}}}$ is the estimation of g, f is suspected in phase with noise-free signal g. ${\text{sign(f) }}$ designate symbolic function.

##### The guided filter

It is found in the experiment, that the B-mode images result found after the application of shrinkage wavelets techniques, still contains noise in the low-frequency component. This result is logical, since shrinkage wavelet makes it possible to retain the wavelet coefficients of the low frequency subband, while the wavelet coefficients of the frequency component are narrowed by the threshold selection. With this in mind, we decided to use a guided filter in order to filter the speckle noise present in the low-frequency component, the researchers opted the guided filter for its effectiveness in preserving the image details [19].

The mathematical form implies a guidance image I, an input image p, and an output image q. Both I and P are given before hand according to the application, and they can be identical. The filtering output at a pixel i is expressed as a weighted average [7]:

3 $${\text{q}}_{\text{i}} = \frac{1}{{\left| {\text{W}} \right|}}\mathop \sum \limits_{{{\text{k}}, {\text{i}} \in {\text{w}}_{\text{k}} }} \left( {{\text{a}}_{\text{k}} {\text{I}}_{\text{i}} + {\text{b}}_{\text{k}} } \right) = {\bar{\text{a}}}_{\text{i}} {\text{I}}_{\text{i}} + {\bar{\text{b}}}_{\text{i}}$$

where q is output image, W represents a function of the guided image I, i is a pixel indexes. Where a_k_ and b_k_ are linear coefficients in w_k_, We assumed that in a window w_k_ centered at the pixel k.

4 $${\bar{\text{a}}}_{\text{i}} = \frac{1}{{\left| {\text{w}} \right|}} \mathop \sum \nolimits {\text{a}}_{\text{k}} \;\quad {\text{and}}\;\quad {\bar{\text{b}}}_{\text{i}} = \frac{1}{{\left| {\text{w}} \right|}} \mathop \sum \nolimits {\text{b}}_{\text{k}}$$

#### Monogenic signal method

After filtering the images of the breast by the shrinkage wavelet technique and the guided filter, we implement them in a monogenic algorithm, the monogenic signal provides the image features [20] energy (A), the local orientation (θ) and phase ($\upvarphi$). These features are computed using two odd filters (Riesz transform) [21].

The monogenic signal has the following format:

5 $${\text{I}}_{\text{M}} \left( {{\text{x}},{\text{y}}} \right) = {\text{p}}\left( {{\text{x}},{\text{y}}} \right) + {\text{iq}}_{1} \left( {{\text{x}},{\text{y}}} \right) + {\text{jq}}_{2} ({\text{x}},{\text{y}})$$

where p(x, y) is the filtered image by using Wavelet shrinkage denoising and guided filter (i, j) are two imaginary components of a quaternion. ${\text{q}}_{1} \left( {{\text{x}},{\text{y}}} \right)\;{\text{and}} \;{\text{q}}_{2} ({\text{x}},{\text{y}})$ represent the result of image filtered with Wavelet shrinkage denoising and guided filter, convolved with Riesz transform.

The following equations give expression of orientation, phase and frequency of its information in a pixel as shown below.

6 $$\uptheta \left( {\text{x}} \right) = \arctan \left( {\frac{{{\text{q}}_{2} \left( {\text{x}} \right)}}{{{\text{q}}_{1} \left( {\text{x}} \right)}}} \right)$$

7 $$\upvarphi \left( {\text{x}} \right) = \arctan \left( {\frac{{|{\text{q}}\left( {\text{x}} \right)|}}{{{\text{p}}({\text{x}})}}} \right)$$

8 $${\text{f}} = {\text{sign}}\left( {\frac{{\updelta_{\upvarphi } }}{{\delta_{\text{x}} }}} \right) \times \sqrt {\left( {\frac{{\updelta_{\upvarphi } }}{{\updelta_{\text{x}} }}} \right)^{2} + \left( {\frac{{\updelta_{\upvarphi } }}{{\delta_{\text{y}} }}} \right)^{2} }$$

According to this, we can create a phase vector if we combine the orientation vector and the phase, formulated along axial displacement (d_x_) and lateral displacement (d_y_).

9 $${\mathbf{r}} = \left( {\upvarphi \cdot \cos \left( \uptheta \right), \;\upvarphi \cdot \sin \left( \uptheta \right)} \right)$$

An analytical estimation of displacement can be obtained, using Taylor series expansions.

The assumption is made as follow:

10 $${\hat{\text{d}}} = \left( {\mathop \sum \limits_{{\mathbf{\aleph }}} \left[ {{\mathbf{n}} \cdot {\mathbf{n}}^{\text{T}} \cdot {\text{f}}} \right]} \right)^{ - 1} \cdot \mathop \sum \limits_{{\mathbf{\aleph }}} \left[ {{\mathbf{r1}} - {\mathbf{r2}}} \right]$$

While (${\aleph }$) is a constant deformation in the region, n is a Taylor series expansion, (**f**) is the frequency, r1 and r2 are two phase vectors along axial and lateral displacement.

## Results

In this section, all results have been verified and validated by two radiologist doctors.

### Soft biological phantom designed for elastography

We implemented the proposed method using Matlab software (The MathWorks, software Matlab, Pentium 4, 3.2 GHz), we tested our method on two models of phantom elasticity sold by the company CIRS, the first phantom contains 10 and 20 mm diameter spheres of varying hardness relative to the background material. The sphere is located at depths of 15 and 35 mm respectively and will appear almost isoechogenic to the background using standard B-mode imaging.

The second phantom contains sets of stepped cylinders that vary in diameter from 1.6 to 16.7 mm. The stepped cylinders in each set are located at depths of 3 and 6 cm. Each set has a different hardness relative to the background material and will appear almost isoechogenic to the background using standard B-mode imaging.

In the software parameterization, we used the linear transducer features with which the phantom was originally acquired: 7.5 MHz center frequency, 60 MHz sampling frequency, 512 physical elements of size 5–0.2 mm (height and width, respectively), with 128 active elements. Two B-mode images (pre-compression and post compression image) are loaded while the pressure was applying by the probe.

**Fig. 1:**
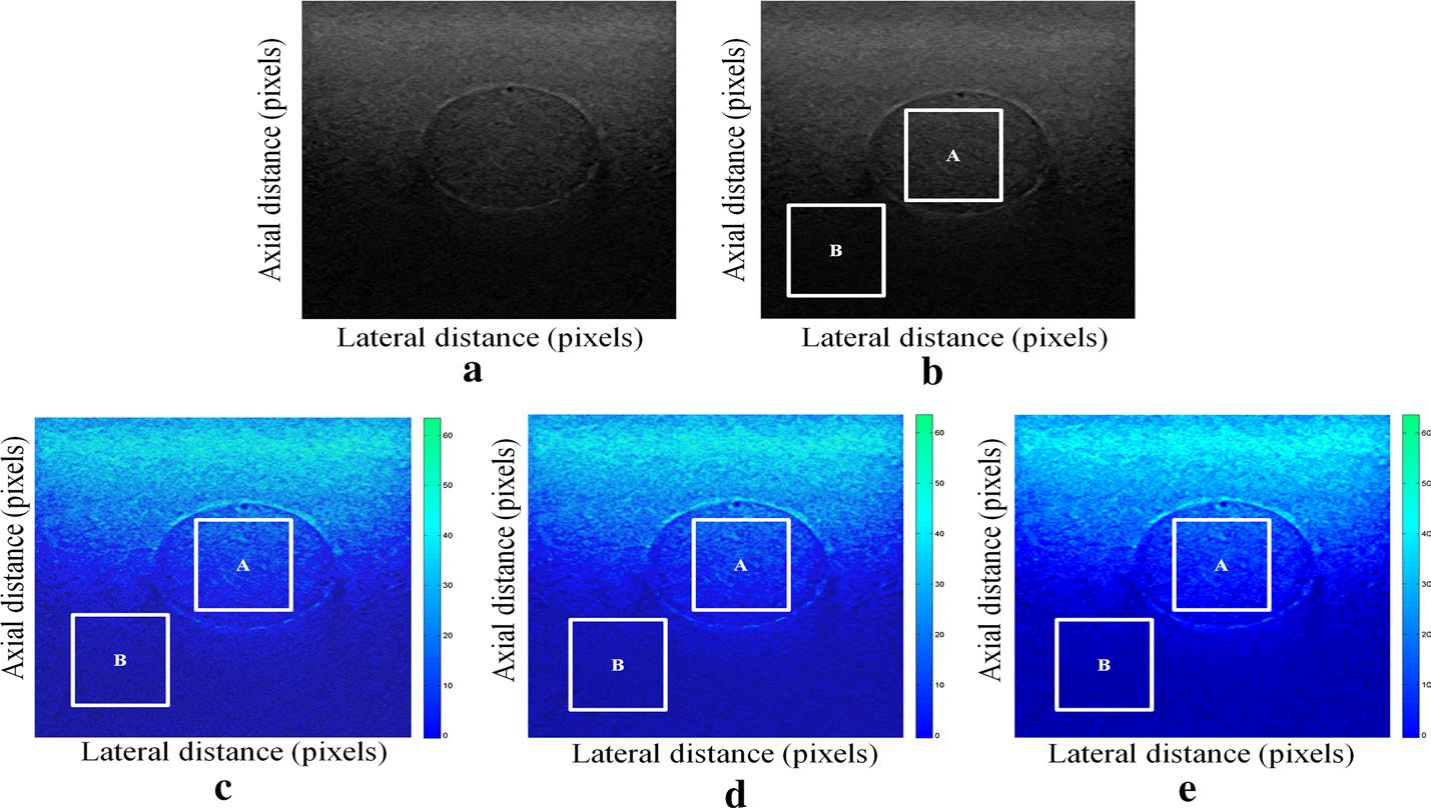
Estimating displacements of a spherical tumor for the first phantom. **a** Simulated pre compression B-mode image. **b** Simulated post compression B-mode image. **c** Tissues displacement obtained with OMS method. **d** tissues displacement obtained with BS method, and **e** tissues displacement obtained with proposed method: areas selected by a *rectangle* are used for CNR computation

**Fig. 2:**
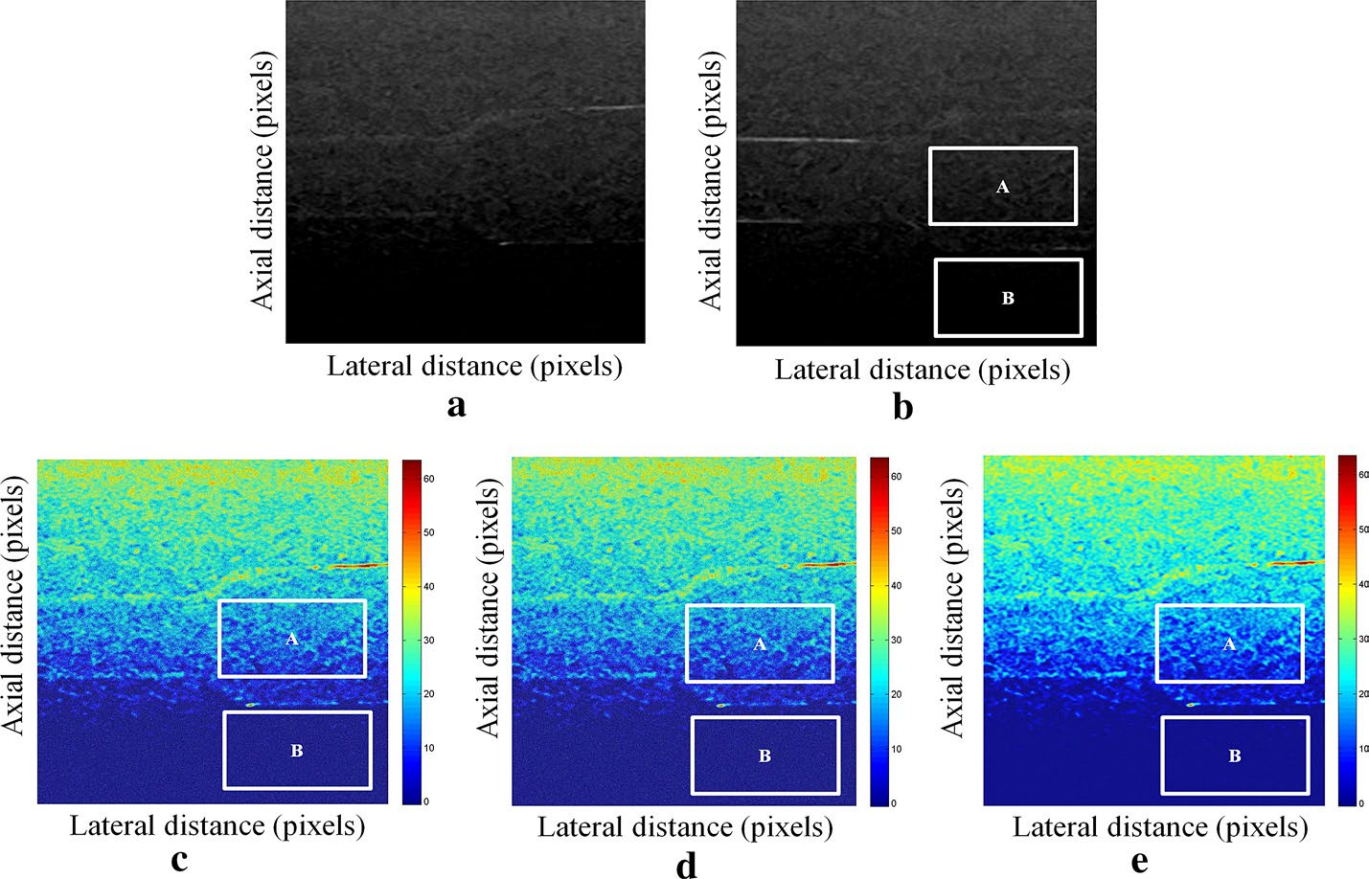
Estimating displacements of a cylindrical tumor for the second phantom. **a** Simulated pre compression B-mode image. **b** Simulated post compression B-mode image. **c** Tissues displacement obtained with OMS method. **d** Tissues displacement obtained with BS method, and **e** tissues displacement obtained with proposed method: areas selected by a *rectangle* are used for CNR computation

We tested our proposed enhancement elastography method on both (pre and after compression B-mode images) and then we implemented them into monogenic signal algorithm to estimate the displacement field. The results were validated by two radiologists (Figs. 1, 2).

In order to quantitatively compare the efficiency and robustness of our proposed method. We compare it to OMS and BS techniques using SD (in pixels) of the errors between estimated and B-mode post compression images (as shown in the Table 1), CNR, PSNR and SSIM comparison (as shown respectively in the Tables 2, 3 and 4), the execution time of each method (as presented in the Tables 5 and 6).

**Table 1:** Comparison of the SD in pixels for the proposed method with OMS and BS methods

	OMS method	BS method	Proposed method
SD in pixels (first phantom)	0.07	0.05	0.03
SD in pixels (second phantom)	15.96	15.80	14.30

**Table 2:** Comparison of CNR for the proposed method with OMS and BS methods

	B-mode image	OMS method	BS method	Proposed method
CNR (first phantom)	0.10	0.29	0.32	0.44
CNR (second phantom)	0.22	0.43	0.48	0.63

**Table 3:** Comparison of PSNR for the proposed method with OMS and BS methods

	OMS method	BS method	Proposed method
PSNR (first phantom)	19	26	33
PSNR (second phantom)	22	31	42

**Table 4:** Comparison of SSIM for the proposed method with OMS and BS methods

	OMS method	BS method	Proposed method
SSIM (first phantom)	0.65	0.69	0.72
SSIM (second phantom)	0.81	0.83	0.87

**Table 5:** Comparison of execution time for the proposed method with OMS and BS methods

	OMS method	BS method	Proposed method
Execution time (s) (first phantom)	12	10	8
Execution time (s) (second phantom)	20	16	12

**Table 6:** Comparison of SDCT for the proposed method with OMS and BS methods

	SDCT between the proposed method and the OMS technique (s)	SDCT between the proposed method and the BS technique (s)
First phantom	4	2
Second phantom	8	4

On these motion phantom results, a CNR is computed as follows:

11 $${\text{CNR}} = \frac{\text{Contrast}}{{{\text{Noise}} }} = \sqrt {\frac{{2({\text{m}}_{\text{b}} - {\text{m}}_{\text{t}} )^{2} }}{{\upsigma_{\text{b}}^{2} + \upsigma_{\text{t}}^{2} }}}$$

where m_t_ and m_b_ are the spatial strain average of the target and background, $\upsigma_{\text{b}}^{2}$ and $\upsigma_{\text{t}}^{2}$ are the spatial strain variance of the target and background, and are the spatial average and variance of a window in the strain image, respectively.

We use the region of interest (ROI) A as the target and ROI B as the background. These parameters (ROI A and ROI B) were used to calculate the CNR and PSNR in the strain images.

The PSNR is defined via the mean squared error (MSE) as:

12 $${\text{MSE}} = \frac{1}{\text{MN}}\mathop \sum \limits_{{{\text{i}} = 1}}^{\text{M}} \mathop \sum \limits_{{{\text{j}} = 1}}^{\text{N}} \left( {{\text{X}}_{\text{i,j}} - {\hat{\text{X}}}_{\text{i,j}} } \right)^{2}$$

where M and N represent the length and width of image corresponding to* X*_*i*,*j*_, its noisy approximation is ${\hat{{X}}}_{{i,j}}$.

13 $${\text{PSNR}}\left( {{\text{X}},{\hat{\text{X}}}} \right) = 10\log \left( {\frac{{255^{2} }}{{{\text{MSE}} }}} \right)$$

The SSIM index is measured between the two windows X and ${\hat{\text{X}}}$ of common length and width:

14 $${\text{SSIM}}\left( {{\text{X}},\,{\hat{\text{X}}}\,} \right) = \frac{{\left( {2\upmu_{\text{X}} \upmu_{{{\hat{\text{X}}}}} + {\text{c}}_{1} } \right)\left( {2\upsigma_{{{\text{X}},{\hat{\text{X}}}}} + {\text{c}}_{2} } \right)}}{{\left( {\upmu_{\text{X}}^{2} + \upmu_{{{\hat{\text{X}}}}}^{2} + {\text{c}}_{1} } \right)\left( {\upsigma_{\text{X}}^{2} + \upsigma_{{{\hat{\text{X}}}}}^{2} + {\text{c}}_{2} } \right)}}$$

where $\upmu_{\text{X}}$, $\upmu_{{{\hat{\text{X}}}}}$, $\upsigma_{\text{x}}^{2}$ and $\upmu_{{{\hat{\text{x}}}}}^{2}$ designate the mean and variance of image and their estimation respectively. $\upsigma_{{{\text{X}},{\hat{\text{X}}}}}$ designates the covariance of X and ${\hat{\text{X}}}$. c_1_ and c_2_ are two variables to maintain the calculation with a low denominator.

### In-vivo breast images

The process described in “Methods” section is assessed here; we use the in vivo breast ultrasound B-mode images (pre and post compression) from 20 patients with breast malignant tumor. Acquired with clinical ultrasound scanner (Logiq E9), with 7.5-MHz linear probe (GE Healthcare).

Both B-mode images (pre and post compression) were taken by the radiologist applying a small compression of the breast, the results were validated by two radiologists.

We have presented below the results of our proposed method for enhancement displacement estimation of breast tissue (Figs. 3, 4, 5, 6, 7, 8, 9, 10, 11, 12, 13, 14, 15, 16, 17, 18, 19, 20, 21 and 22).

**Fig. 3:**
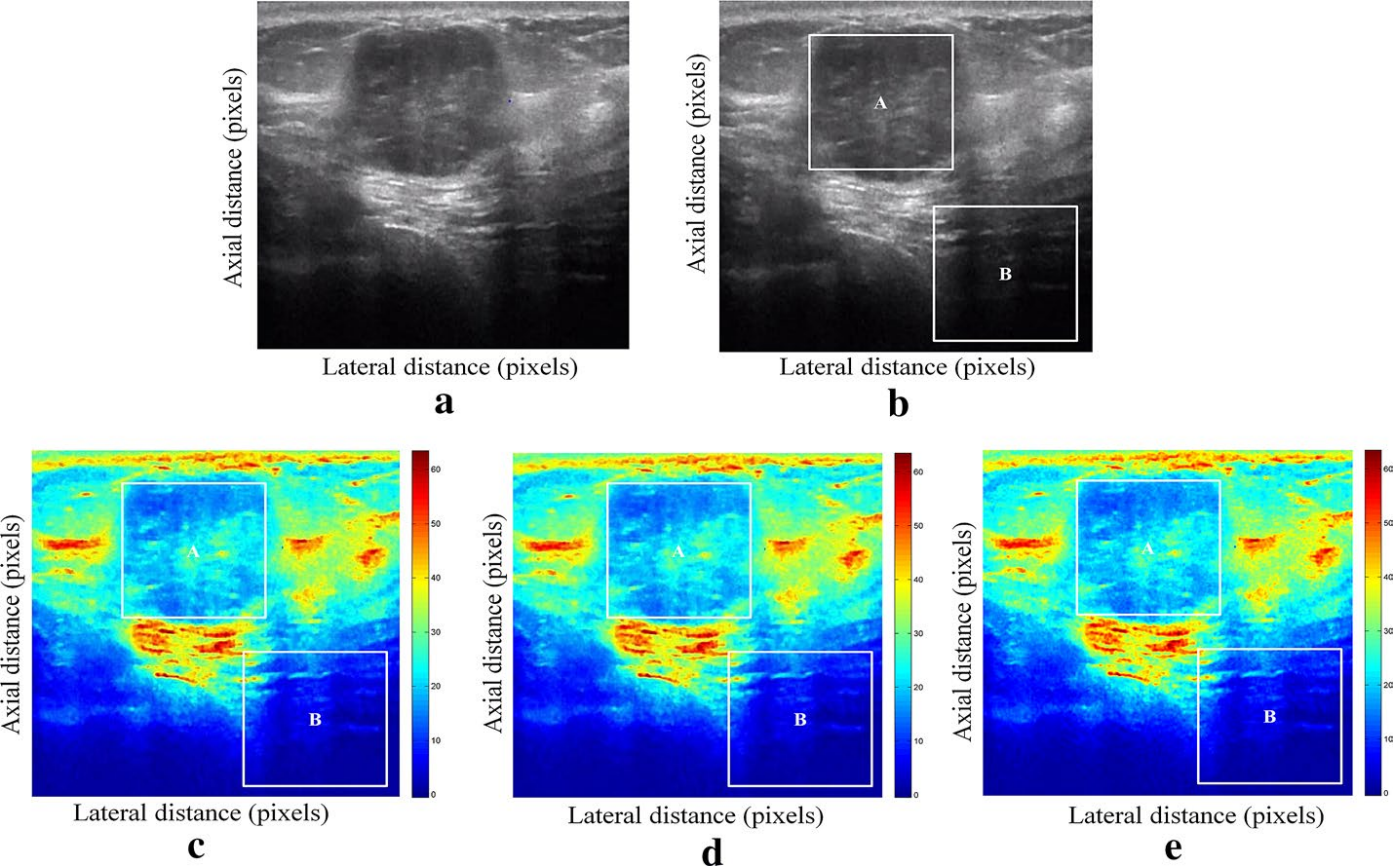
Estimating tissue displacements of breast malignant tumor for the patient 1. **a** Simulated pre compression B-mode image. **b** Simulated post compression B-mode image. **c** Tissues displacement obtained with OMS method. **d** Tissues displacement obtained with BS method, and **e** tissues displacement obtained with proposed method: areas selected by a *rectangle* are used for CNR computation

**Fig. 4:**
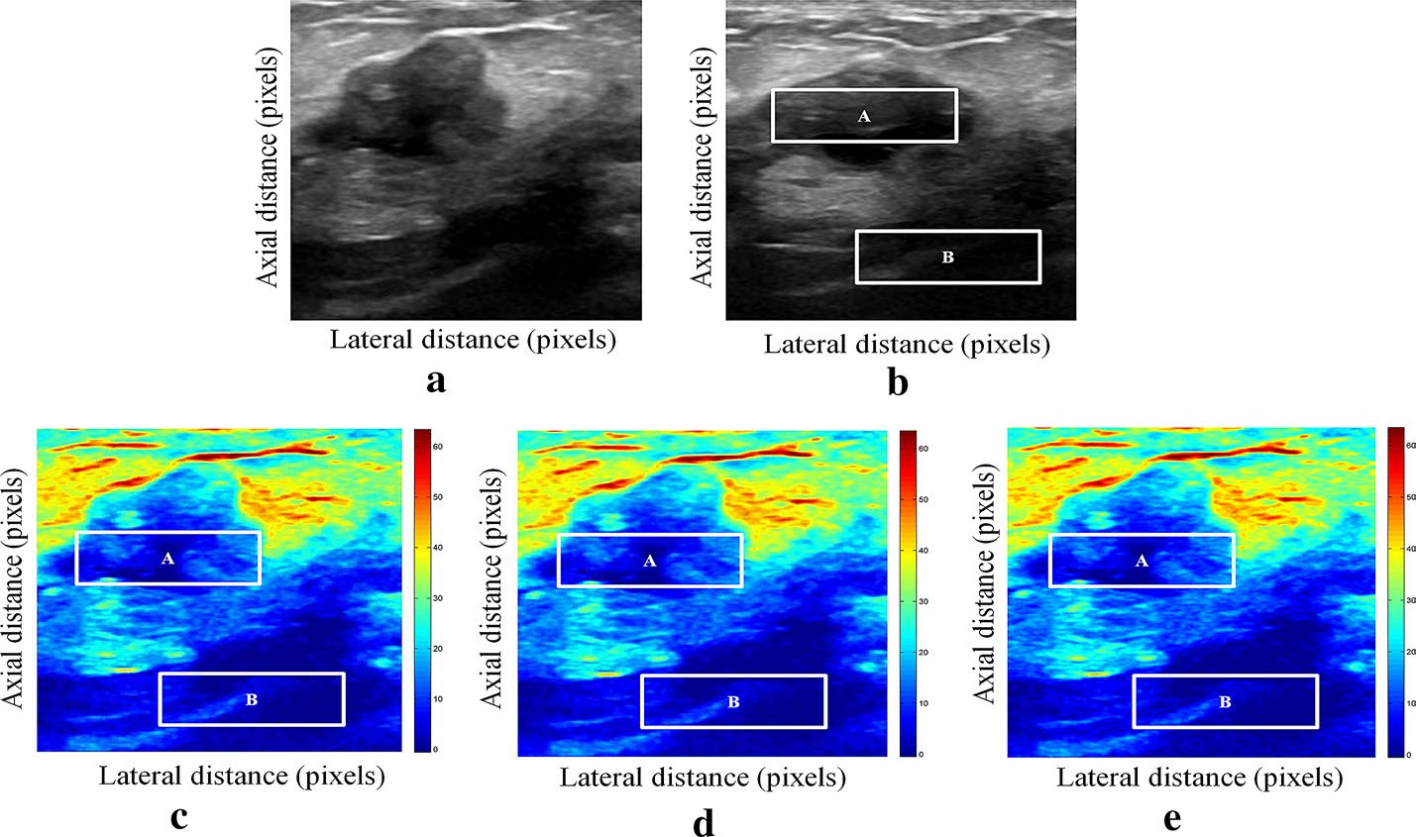
Estimating tissue displacements of breast malignant tumor for the patient 2. **a** Simulated pre compression B-mode image. **b** Simulated post compression B-mode image. **c** Tissues displacement obtained with OMS method. **d** Tissues displacement obtained with BS method, and **e** tissues displacement obtained with proposed method: areas selected by a *rectangle* are used for CNR computation

**Fig. 5:**
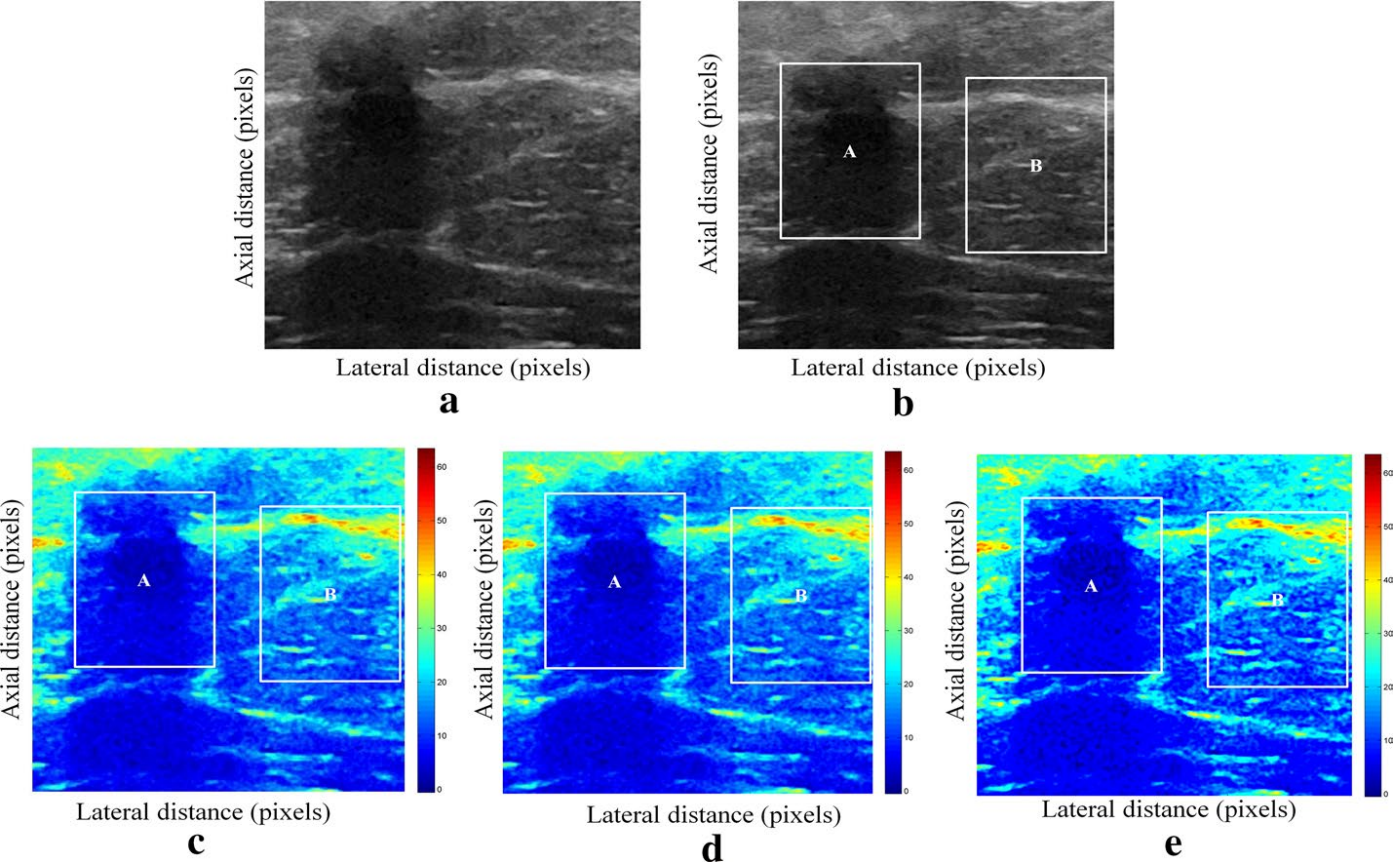
Estimating tissue displacements of breast malignant tumor for the patient 3. **a** Simulated pre compression B-mode image. **b** Simulated post compression B-mode image. **c** Tissues displacement obtained with OMS method. **d** Tissues displacement obtained with BS method, and **e** tissues displacement obtained with proposed method: areas selected by a *rectangle* are used for CNR computation

**Fig. 6:**
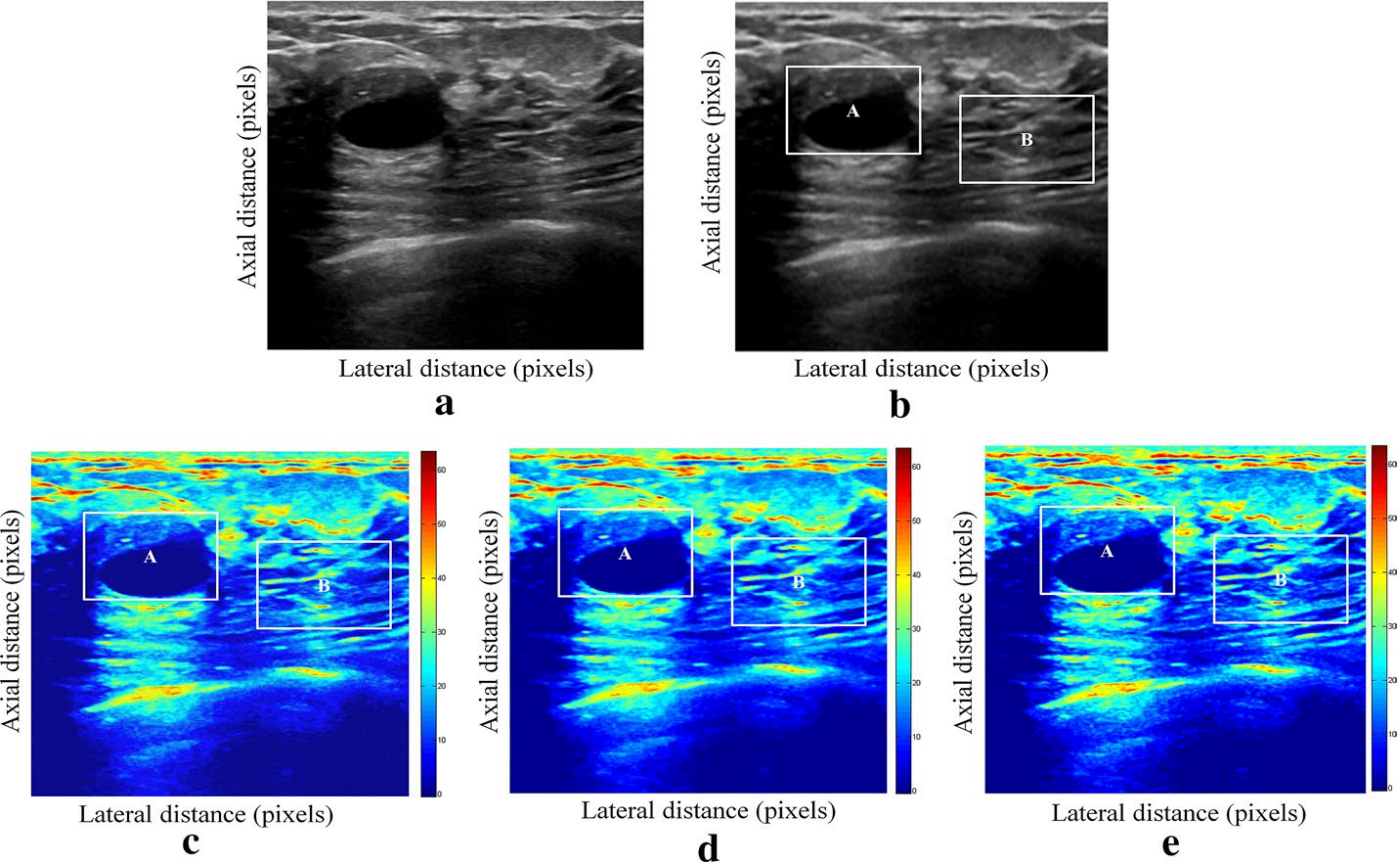
Estimating tissue displacements of breast malignant tumor for the patient 4. **a** Simulated pre compression B-mode image. **b** Simulated post compression B-mode image. **c** Tissues displacement obtained with OMS method. **d** Tissues displacement obtained with BS method, and **e** tissues displacement obtained with proposed method: areas selected by a *rectangle* are used for CNR computation

**Fig. 7:**
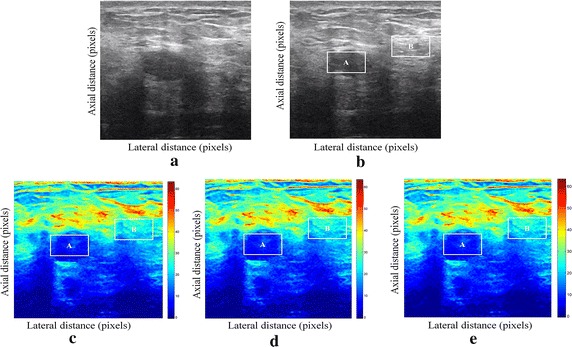
Estimating tissue displacements of breast malignant tumor for the patient 5. **a** Simulated pre compression B-mode image. **b** Simulated post compression B-mode image. **c** Tissues displacement obtained with OMS method. **d** Tissues displacement obtained with BS method, and **e** tissues displacement obtained with proposed method: areas selected by a *rectangle* are used for CNR computation

**Fig. 8:**
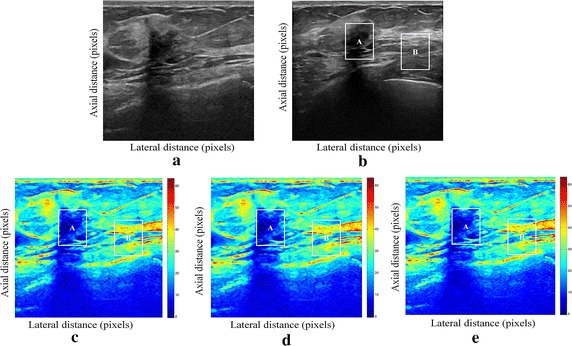
Estimating tissue displacements of breast malignant tumor for the patient 6. **a** Simulated pre compression B-mode image. **b** Simulated post compression B-mode image. **c** Tissues displacement obtained with OMS method. **d** Tissues displacement obtained with BS method, and **e** tissues displacement obtained with proposed method: areas selected by a *rectangle* are used for CNR computation

**Fig. 9:**
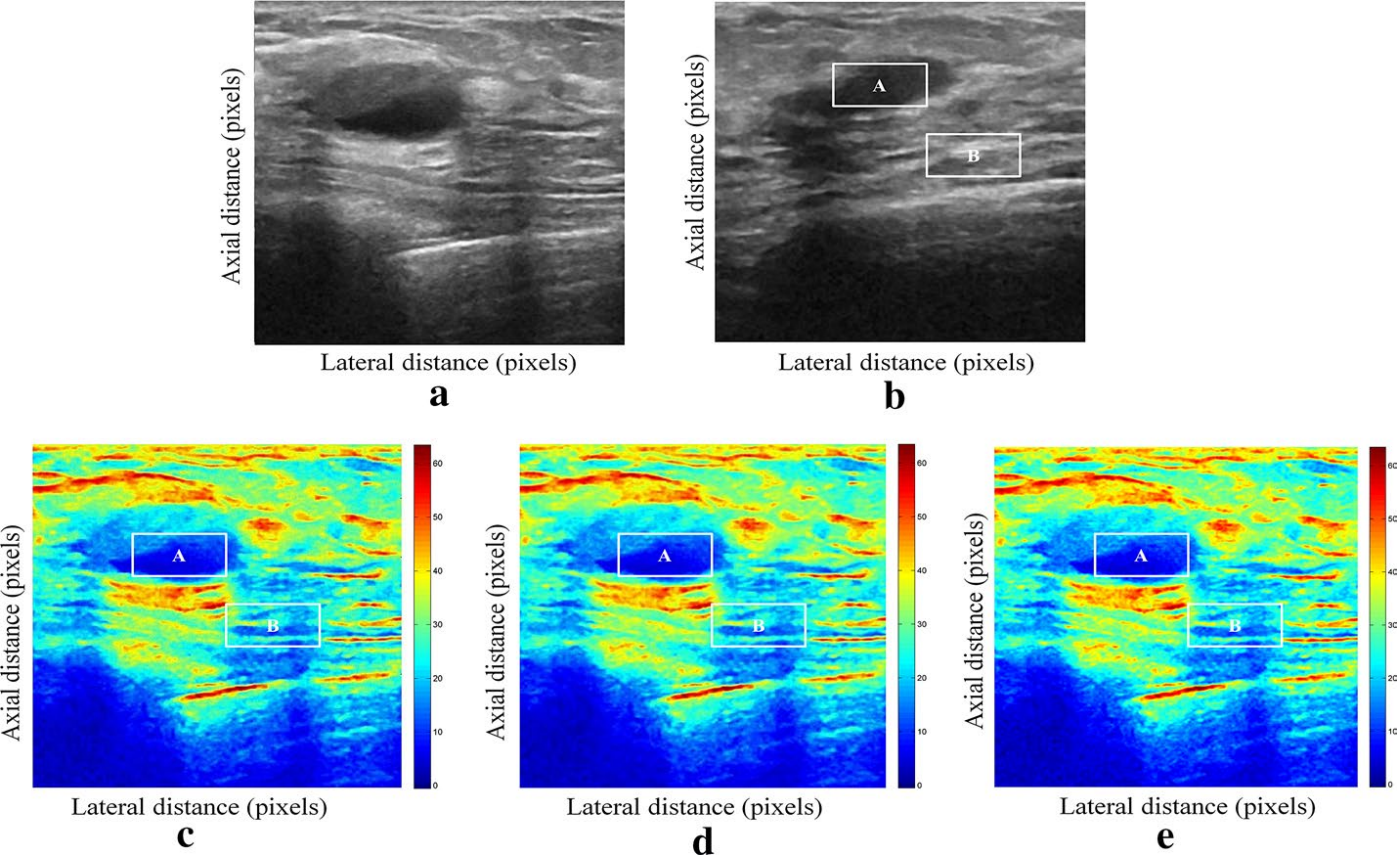
Estimating tissue displacements of breast malignant tumor for the patient 7. **a** Simulated pre compression B-mode image. **b** Simulated post compression B-mode image. **c** Tissues displacement obtained with OMS method. **d** Tissues displacement obtained with BS method, and **e** tissues displacement obtained with proposed method: areas selected by a *rectangle* are used for CNR computation

**Fig. 10:**
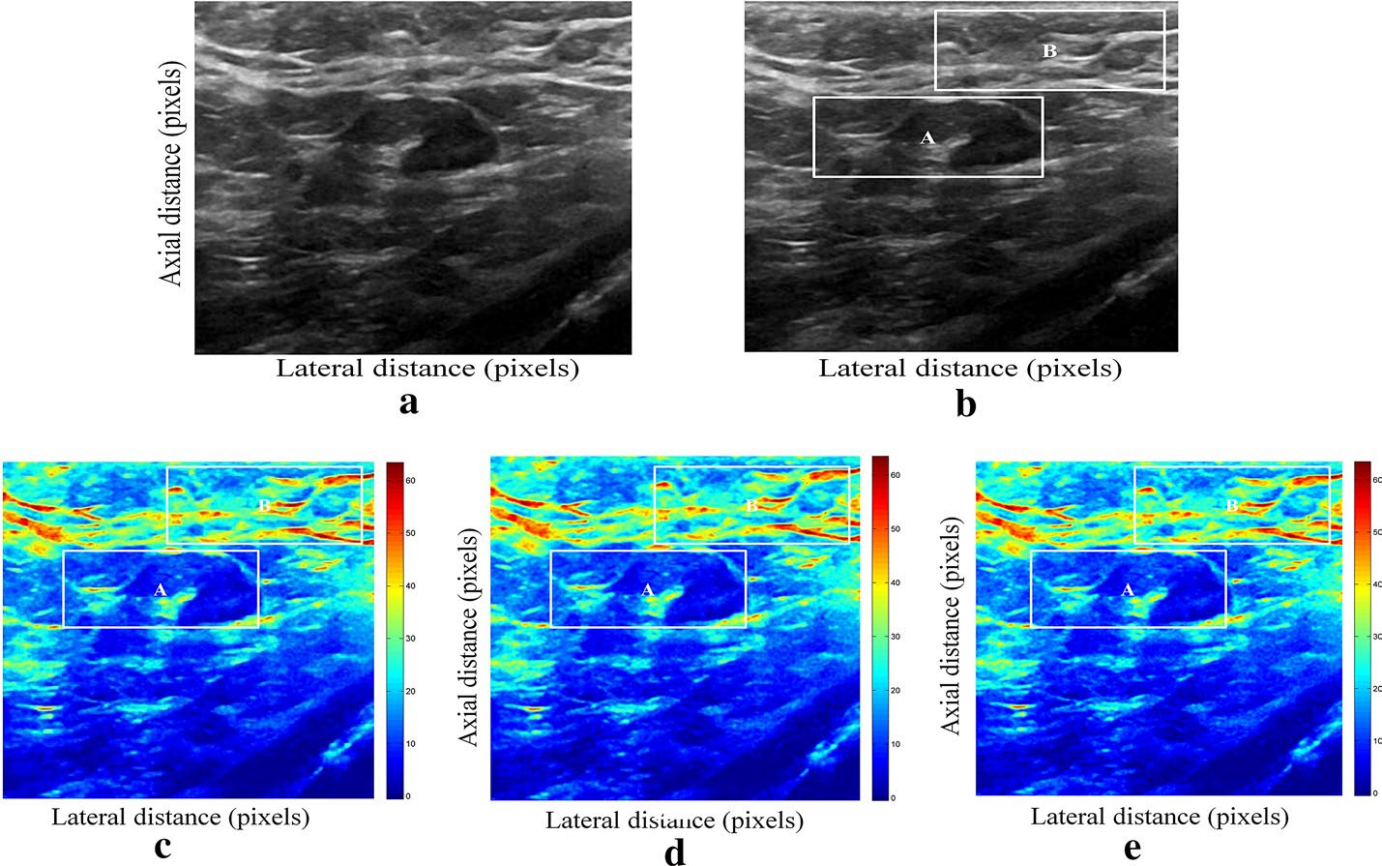
Estimating tissue displacements of breast malignant tumor for the patient 8. **a** Simulated pre compression B-mode image. **b** Simulated post compression B-mode image. **c** Tissues displacement obtained with OMS method. **d** Tissues displacement obtained with BS method, and **e** tissues displacement obtained with proposed method: areas selected by a *rectangle* are used for CNR computation

**Fig. 11:**
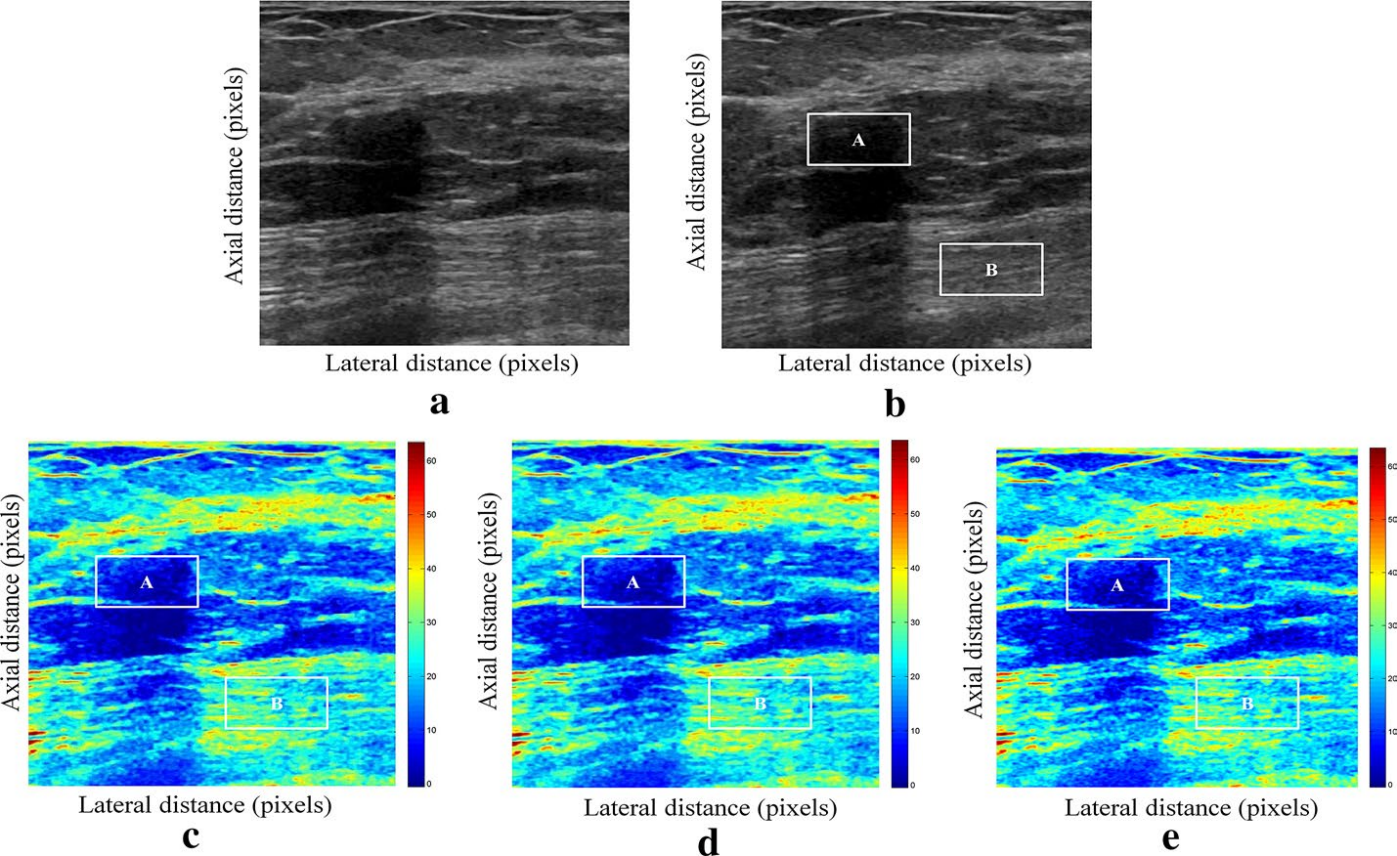
Estimating tissue displacements of breast malignant tumor for the patient 9. **a** Simulated pre compression B-mode image. **b** Simulated post compression B-mode image. **c** Tissues displacement obtained with OMS method. **d** Tissues displacement obtained with BS method, and **e** tissues displacement obtained with proposed method: areas selected by a *rectangle* are used for CNR computation

**Fig. 12:**
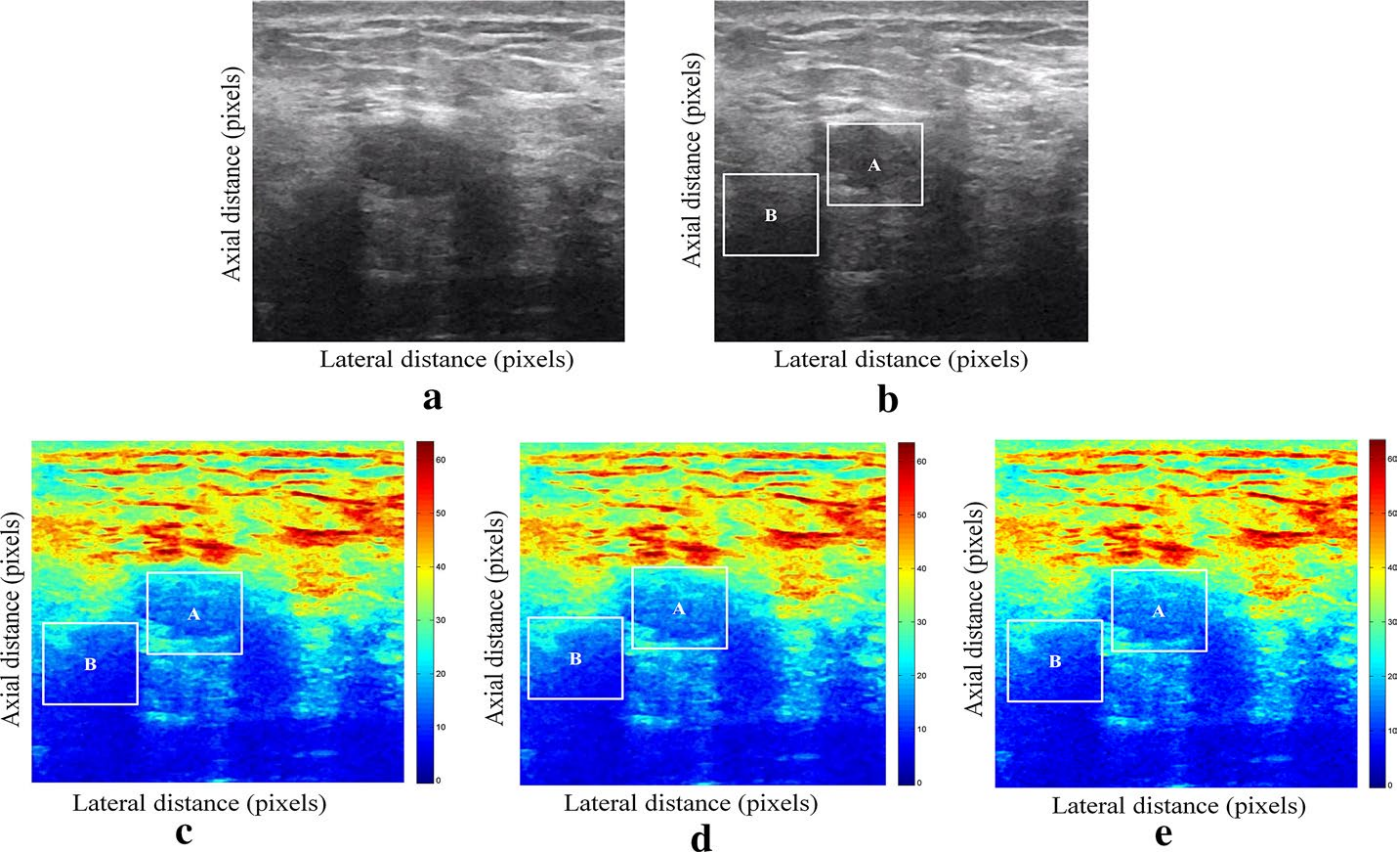
Estimating tissue displacements of breast malignant tumor for the patient 10. **a** Simulated pre compression B-mode image. **b** Simulated post compression B-mode image. **c** Tissues displacement obtained with OMS method. **d** Tissues displacement obtained with BS method, and **e** tissues displacement obtained with proposed method: areas selected by a *rectangle* are used for CNR computation

**Fig. 13:**
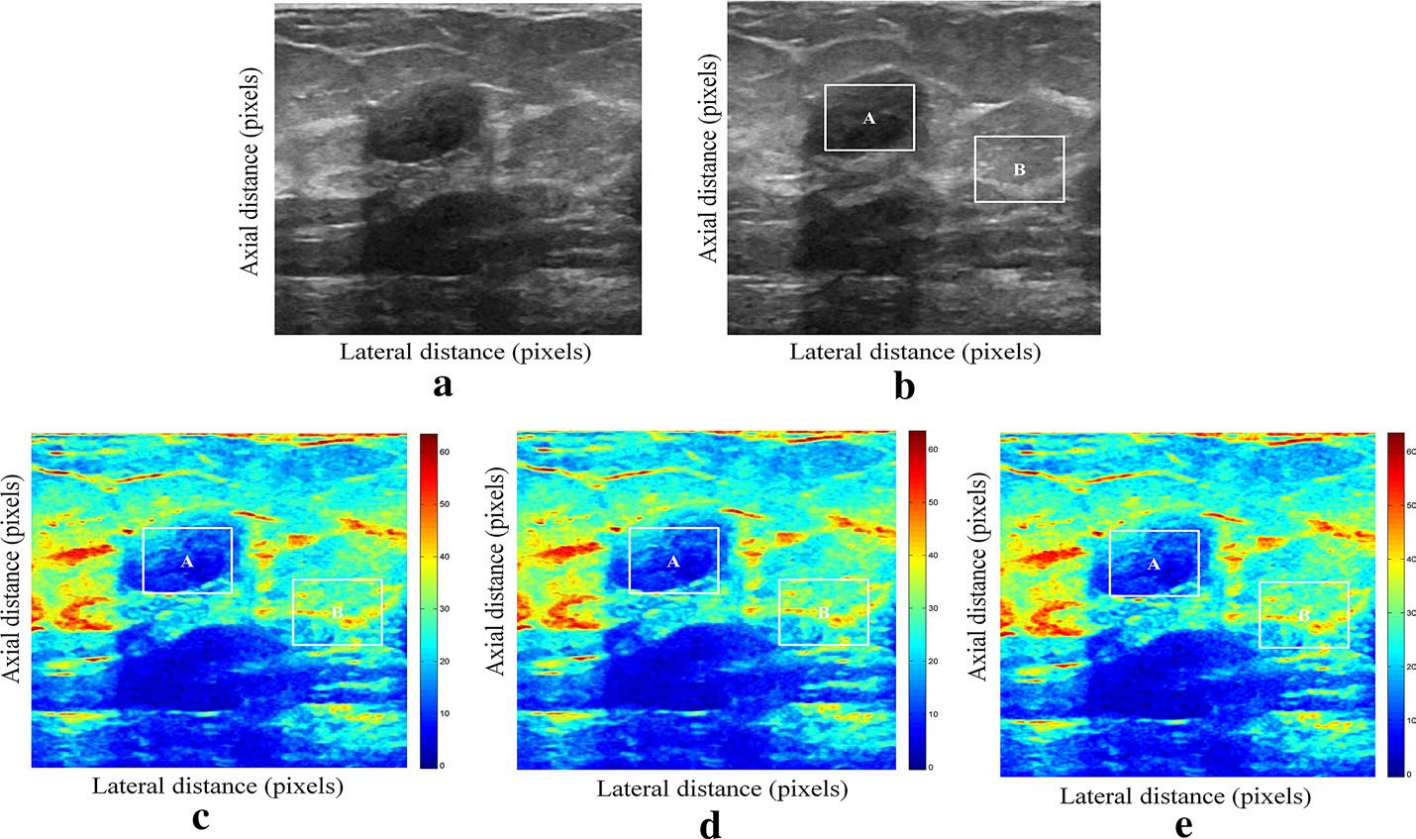
Estimating tissue displacements of breast malignant tumor for the patient 11. **a** Simulated pre compression B-mode image. **b** Simulated post compression B-mode image. **c** Tissues displacement obtained with OMS method. **d** Tissues displacement obtained with BS method, and **e** tissues displacement obtained with proposed method: areas selected by a *rectangle* are used for CNR computation

**Fig. 14:**
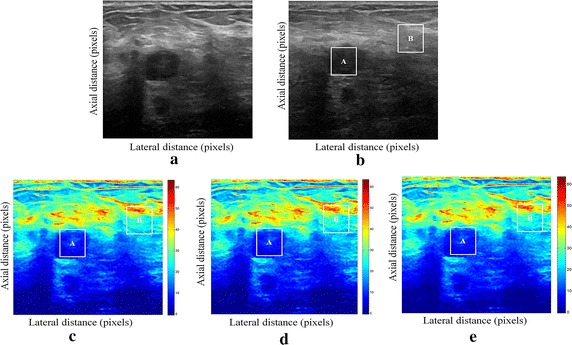
Estimating tissue displacements of breast malignant tumor for the patient 12. **a** Simulated pre compression B-mode image. **b** Simulated post compression B-mode image. **c** Tissues displacement obtained with OMS method. **d** Tissues displacement obtained with BS method, and **e** tissues displacement obtained with proposed method: areas selected by a *rectangle* are used for CNR computation

**Fig. 15:**
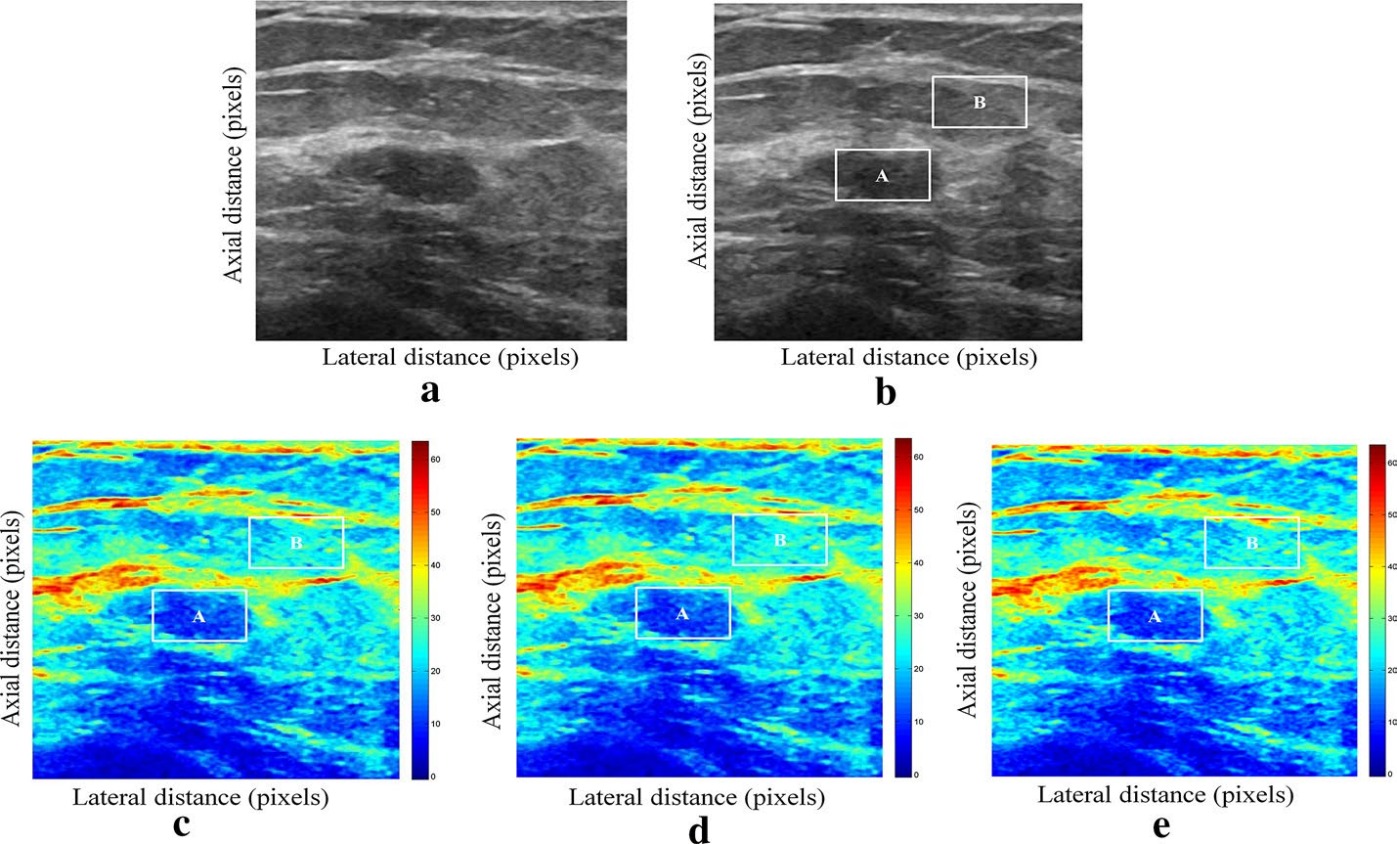
Estimating tissue displacements of breast malignant tumor for the patient 13. **a** Simulated pre compression B-mode image. **b** Simulated post compression B-mode image. **c** Tissues displacement obtained with OMS method. **d** Tissues displacement obtained with BS method, and **e** tissues displacement obtained with proposed method: areas selected by a *rectangle* are used for CNR computation

**Fig. 16:**
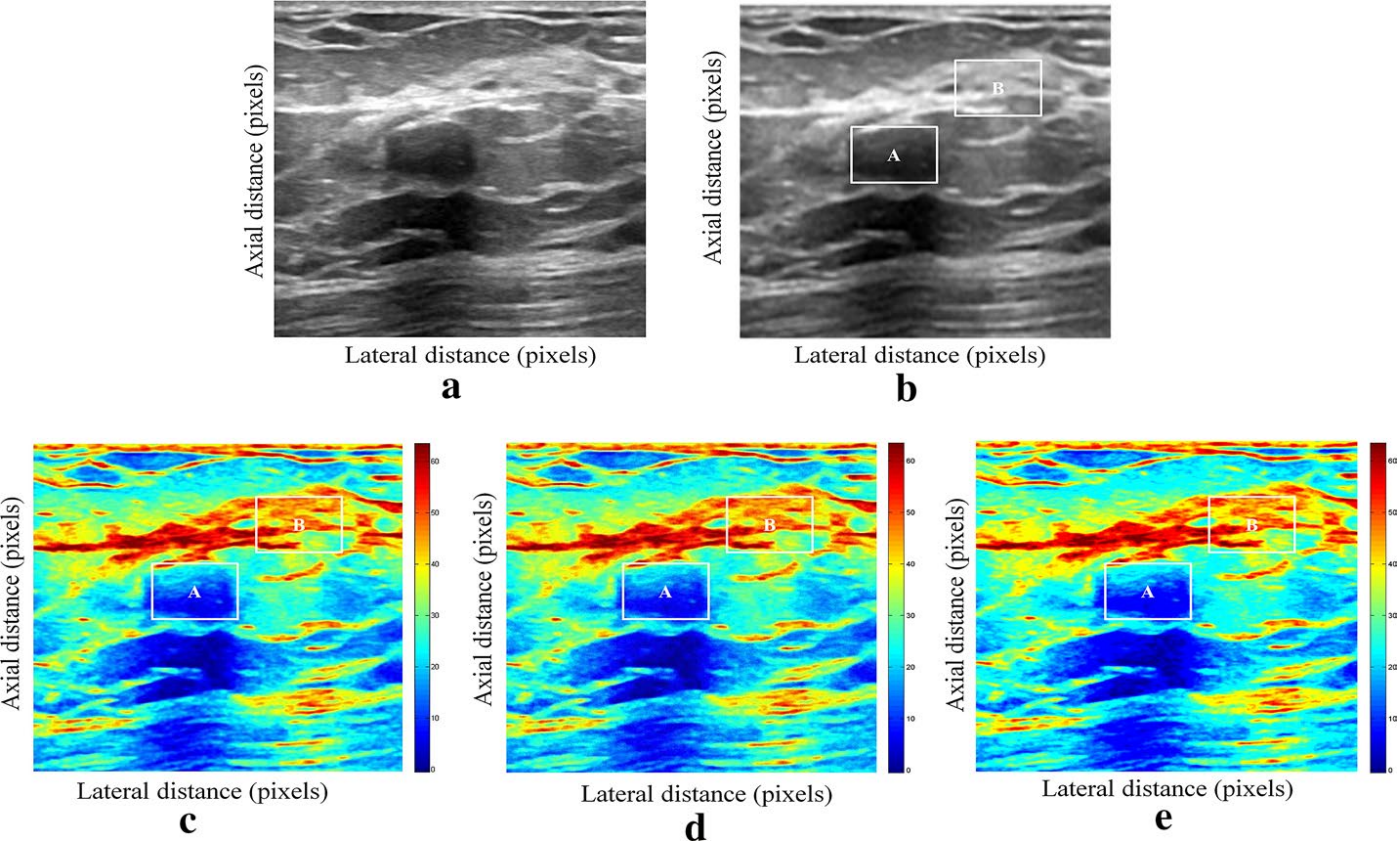
Estimating tissue displacements of breast malignant tumor for the patient 14. **a** Simulated pre compression B-mode image. **b** Simulated post compression B-mode image. **c** Tissues displacement obtained with OMS method. **d** Tissues displacement obtained with BS method, and **e** tissues displacement obtained with proposed method: areas selected by a *rectangle* are used for CNR computation

**Fig. 17:**
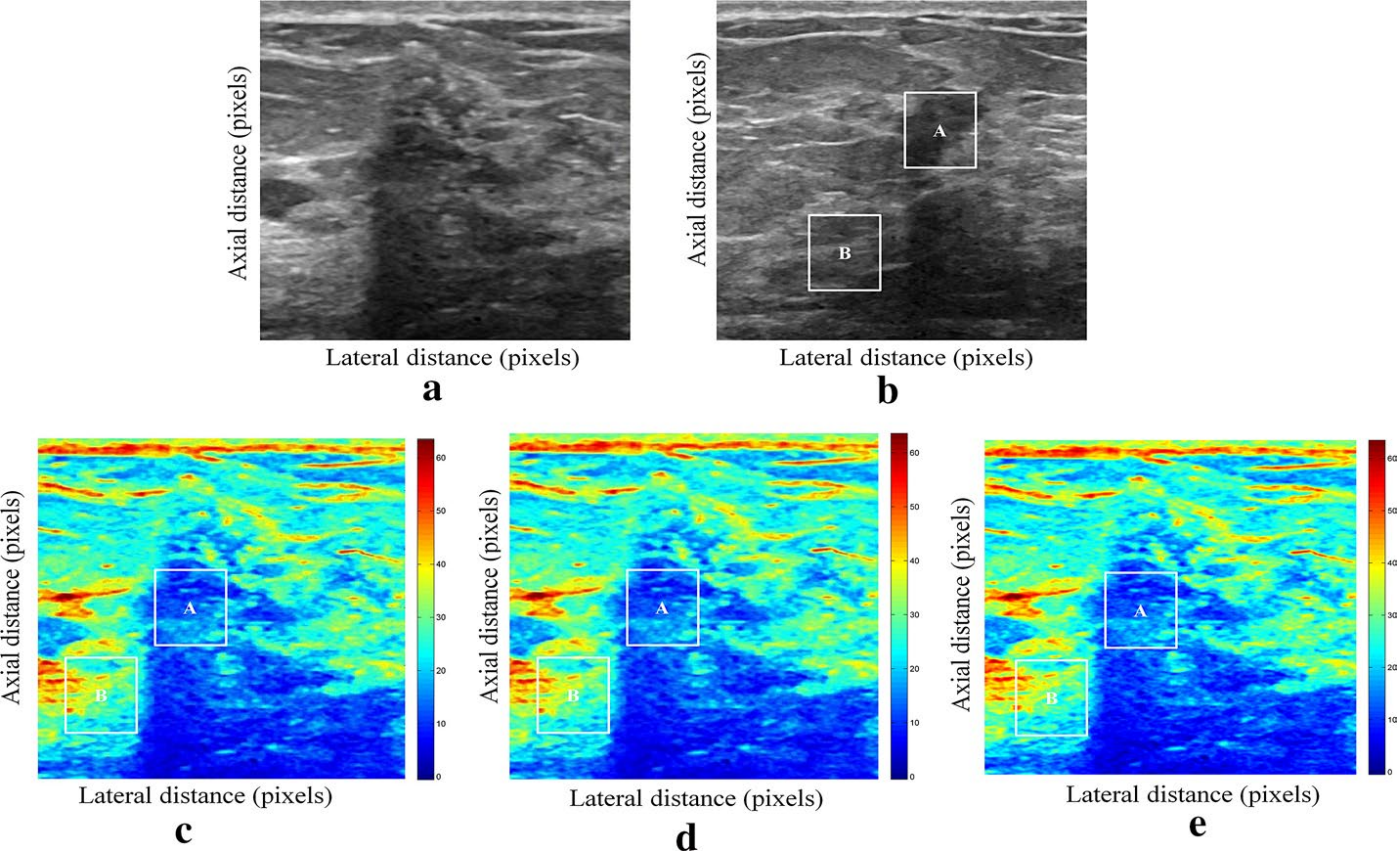
Estimating tissue displacements of breast malignant tumor for the patient 15. **a** Simulated pre compression B-mode image. **b** Simulated post compression B-mode image. **c** Tissues displacement obtained with OMS method. **d** Tissues displacement obtained with BS method, and **e** tissues displacement obtained with proposed method: areas selected by a *rectangle* are used for CNR computation

**Fig. 18:**
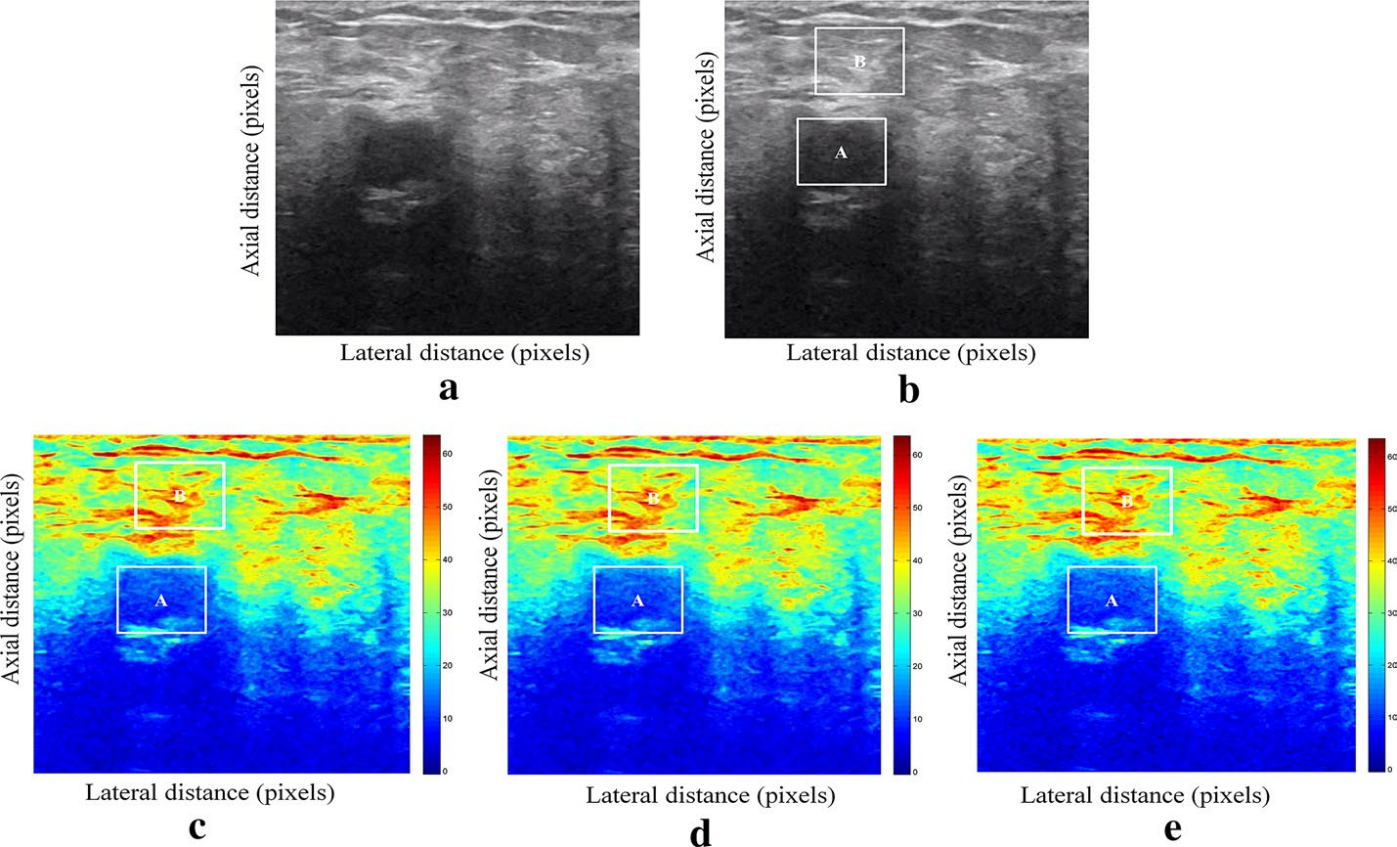
Estimating tissue displacements of breast malignant tumor for the patient 16. **a** Simulated pre compression B-mode image. **b** Simulated post compression B-mode image. **c** Tissues displacement obtained with OMS method. **d** Tissues displacement obtained with BS method, and **e** tissues displacement obtained with proposed method: areas selected by a *rectangle* are used for CNR computation

**Fig. 19:**
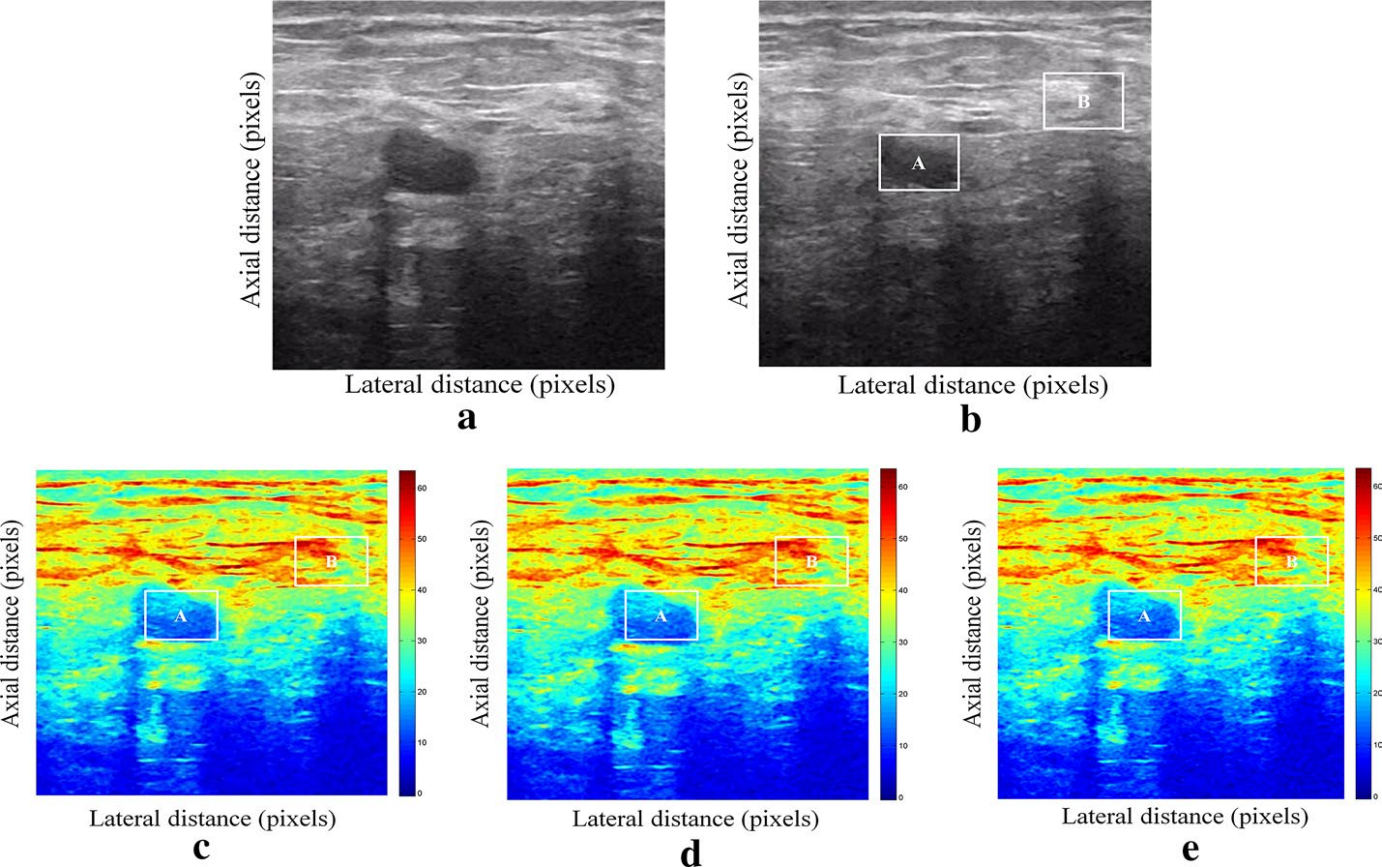
Estimating tissue displacements of breast malignant tumor for the patient 17. **a** Simulated pre compression B-mode image. **b** Simulated post compression B-mode image. **c** Tissues displacement obtained with OMS method. **d** Tissues displacement obtained with BS method, and **e** tissues displacement obtained with proposed method: areas selected by a *rectangle* are used for CNR computation

**Fig. 20:**
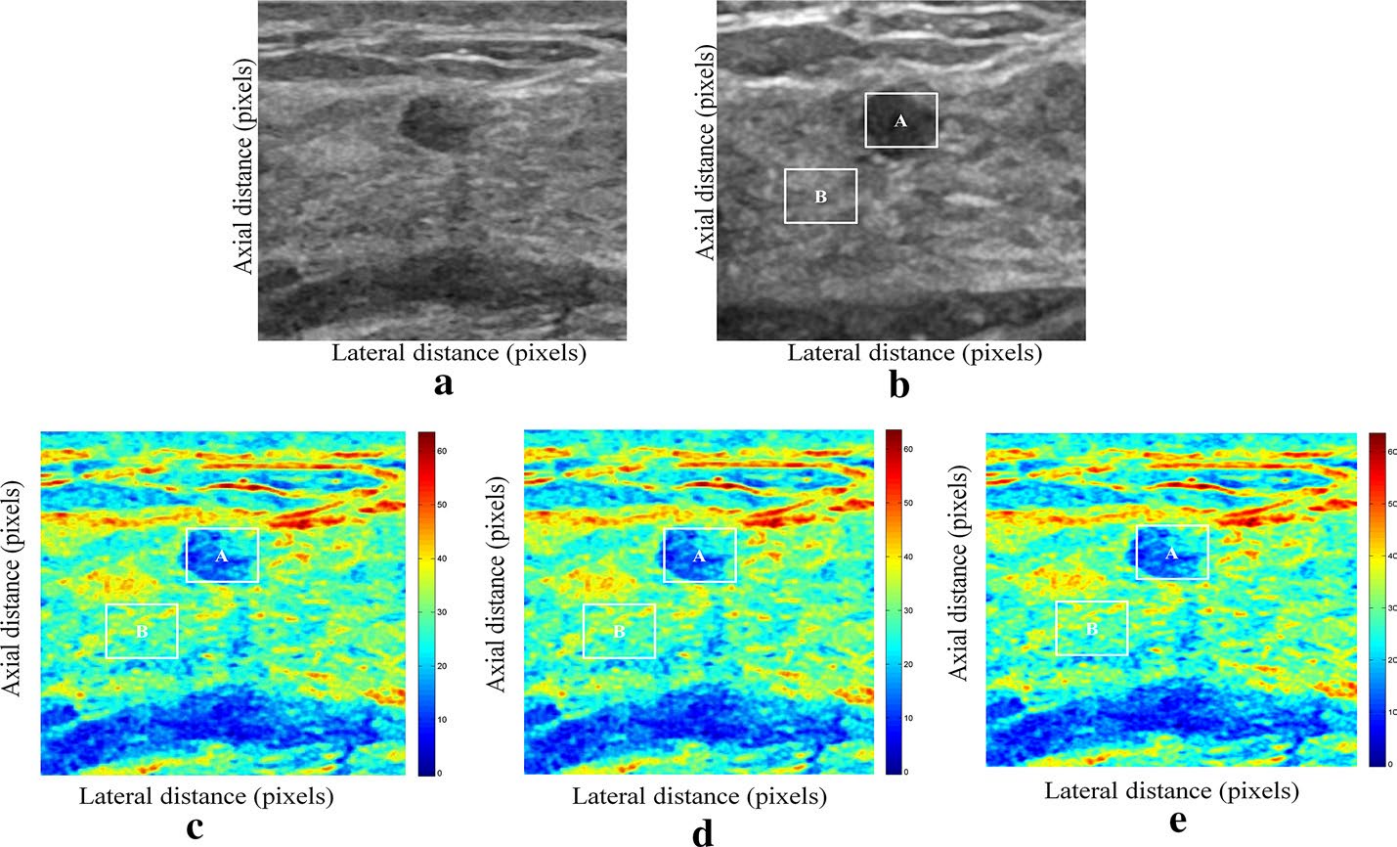
Estimating tissue displacements of breast malignant tumor for the patient 18. **a** Simulated pre compression B-mode image. **b** Simulated post compression B-mode image. **c** Tissues displacement obtained with OMS method. **d** Tissues displacement obtained with BS method, and **e** tissues displacement obtained with proposed method: areas selected by a *rectangle* are used for CNR computation

**Fig. 21:**
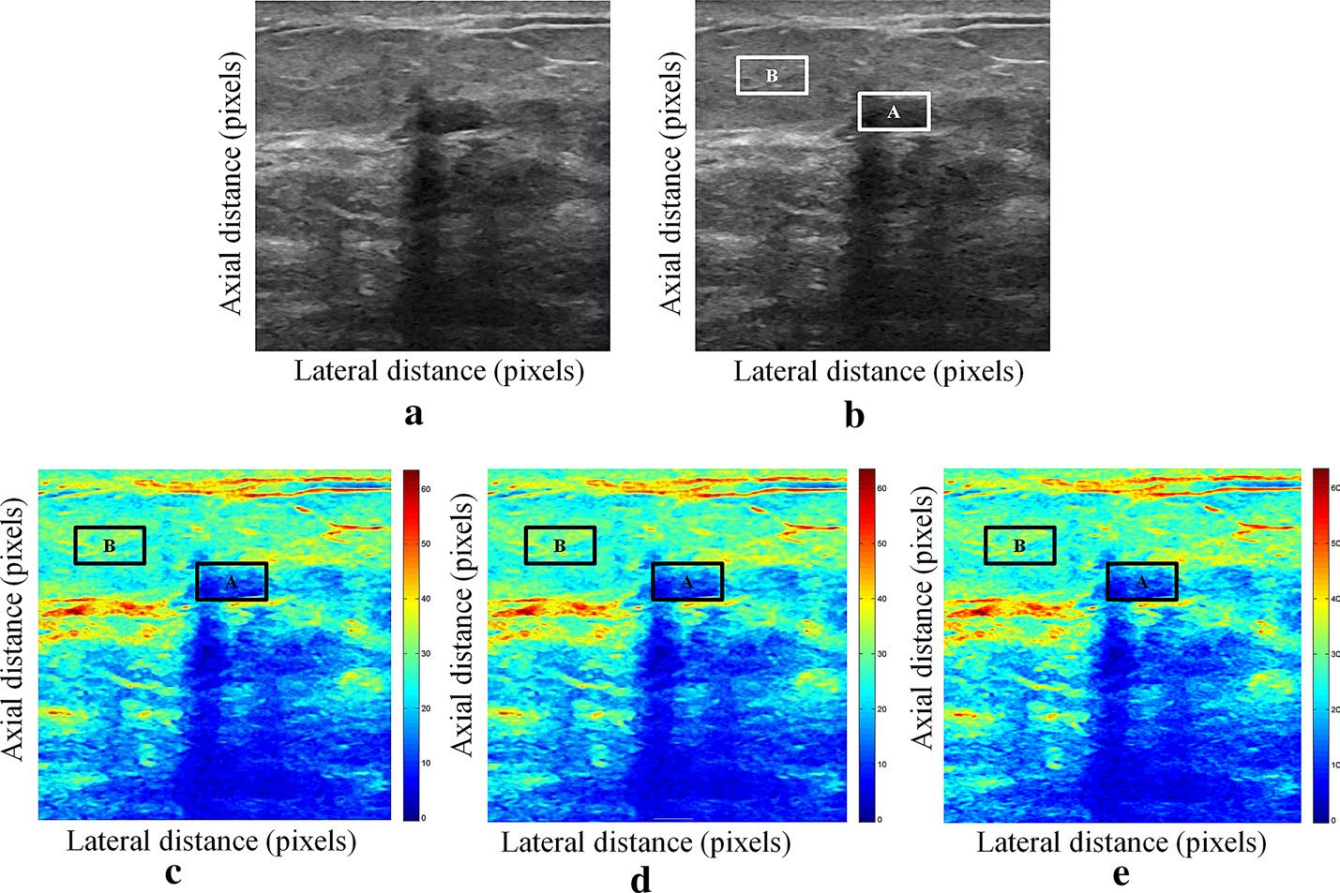
Estimating tissue displacements of breast malignant tumor for the patient 19. **a** Simulated pre compression B-mode image. **b** Simulated post compression B-mode image. **c** Tissues displacement obtained with OMS method. **d** Tissues displacement obtained with BS method, and **e** tissues displacement obtained with proposed method: areas selected by a *rectangle* are used for CNR computation

**Fig. 22:**
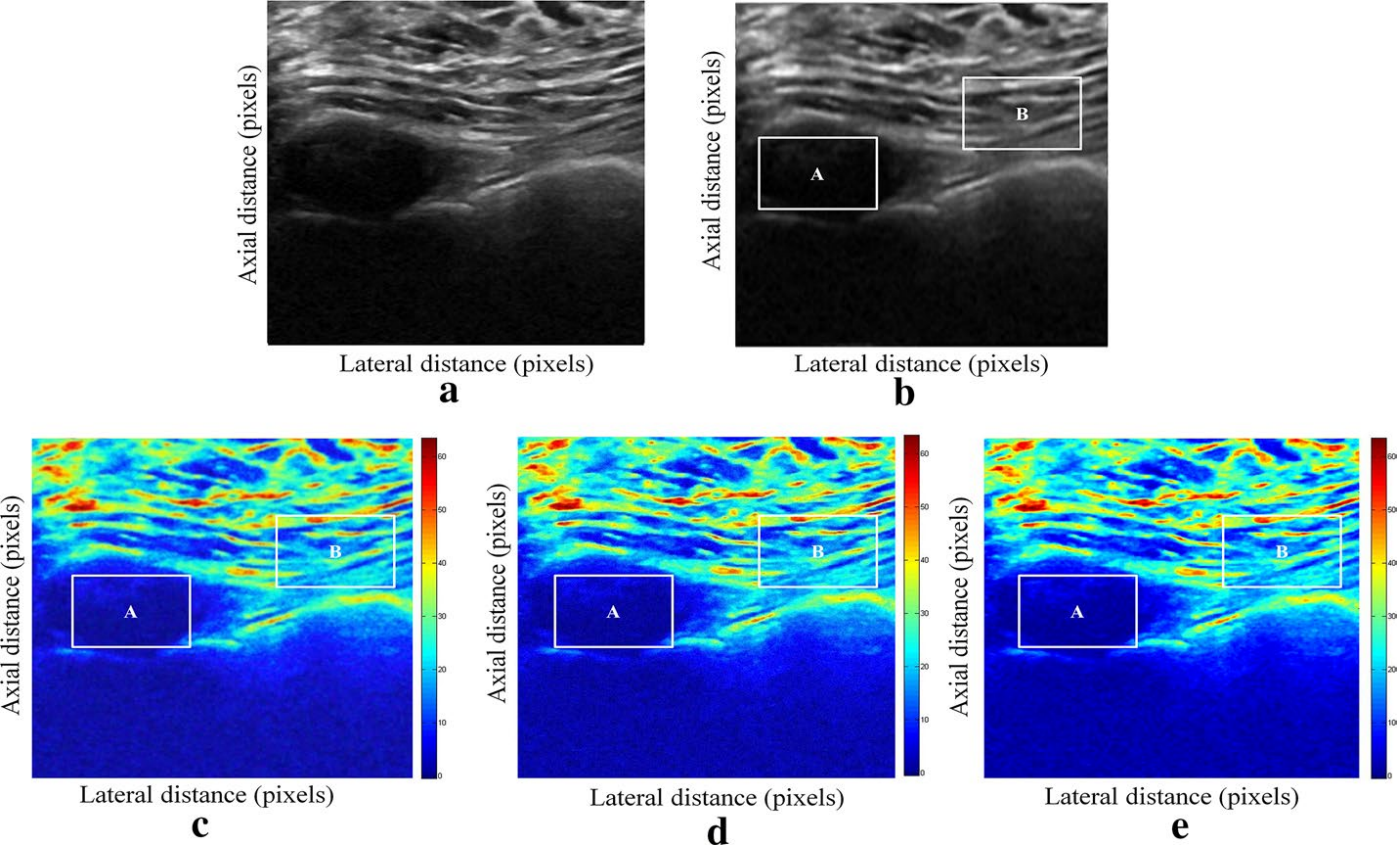
Estimating tissue displacements of breast malignant tumor for the patient 20. **a** Simulated pre compression B-mode image. **b** Simulated post compression B-mode image. **c** tissues displacement obtained with OMS method. **d** tissues displacement obtained with BS method, and **e** tissues displacement obtained with proposed method: areas selected by a *rectangle* are used for CNR computation

In order to evaluate the efficiency of our approach, quantitative measures as SD in pixels (as shown in the Table 7), CNR comparison (as presented in the Table 8), PSNR comparison (as given in the Table 9), SSIM (as demonstrated in the Table 10), the execution time of each method (as exposed in the Table 11) and the SDCT (as shown in the Table 12) were used in order to highlight the contribution of our method and to compare its accuracy with OMS and BS approaches.

**Table 7:** In vivo results comparison of SD in pixels for the proposed method with OMS and BS methods for 20 patients

	OMS method	BS method	Proposed method
SD in pixels: patient 1	5.88	5.28	4.98
SD in pixels: patient 2	4.76	4.11	3.92
SD in pixels: patient 3	3.87	3.27	2.87
SD in pixels: patient 4	8.32	7.98	6.54
SD in pixels: patient 5	6.20	4.76	3.55
SD in pixels: patient 6	8.98	8.21	7.98
SD in pixels: patient 7	4.87	3.68	2.43
SD in pixels: patient 8	3.01	2.97	2.15
SD in pixels: patient 9	4.65	3.93	2.84
SD in pixels: patient 10	3.10	2.31	2.11
SD in pixels: patient 11	5.85	4.97	3.48
SD in pixels: patient 12	7.43	5.87	4.75
SD in pixels: patient 13	8.09	6.21	5.89
SD in pixels: patient 14	4.72	4.65	4.12
SD in pixels: patient 15	3.52	2.47	2.33
SD in pixels: patient 16	5	4.71	4.53
SD in pixels: patient 17	6.18	4.81	3.76
SD in pixels: patient 18	8.31	6.93	5.92
SD in pixels: patient 19	7.16	5.24	4.51
SD in pixels: patient 20	5.41	3.64	3.02

**Table 8:** Comparison of CNR for the proposed method with OMS and BS methods for 20 patients

	B-mode image	OMS method	BS method	Proposed method
CNR: patient 1	0.31	0.39	0.41	0.62
CNR: patient 2	1.62	1.97	2.01	2.32
CNR: patient 3	0.62	1.12	1.20	1.27
CNR: patient 4	1.14	1.98	2.03	2.47
CNR: patient 5	0.74	1.31	1.40	1.76
CNR: patient 6	0.16	0.93	1.21	1.43
CNR: patient 7	1.23	1.76	1.87	1.97
CNR: patient 8	0.18	0.86	0.92	1.16
CNR: patient 9	0.95	1.32	1.99	2.23
CNR: patient 10	0.19	0.49	0.52	1.23
CNR: patient 11	0.53	0.97	1.12	1.45
CNR: patient 12	0.61	1.32	1.63	1.97
CNR: patient 13	0.73	1.83	1.98	2.31
CNR: patient 14	0.39	0.84	0.89	1.36
CNR: patient 15	0.41	0.79	0.96	1.42
CNR: patient 16	0.72	1.14	1.38	1.79
CNR: patient 17	0.87	1.34	1.64	1.84
CNR: patient 18	0.74	1.20	1.56	1.95
CNR: patient 19	0.73	1.17	1.53	2.09
CNR: patient 20	0.39	1.16	1.37	1.79

**Table 9:** Comparison of PSNR for the proposed method with OMS and BS methods for 20 patients

	OMS method	BS method	Proposed method
PSNR: patient 1	23.95	26.17	29.31
PSNR: patient 2	12.80	15.30	17.82
PSNR: patient 3	24.87	31.02	34.91
PSNR: patient 4	15.83	17.21	19.78
PSNR: patient 5	37.85	42.80	44.71
PSNR: patient 6	14.36	16.21	20.31
PSNR: patient 7	30.96	37.31	38.43
PSNR: patient 8	27.84	32.80	37.96
PSNR: patient 9	37.53	39.12	41.02
PSNR: patient 10	29.31	34.91	36.17
PSNR: patient 11	18.98	20.93	25.95
PSNR: patient 12	32.61	38.59	44.18
PSNR: patient 13	12.44	14.71	17.50
PSNR: patient 14	39.42	41.60	43.11
PSNR: patient 15	43.04	46.13	52.64
PSNR: patient 16	23.80	27.07	32.85
PSNR: patient 17	19.71	22.95	23.96
PSNR: patient 18	23.05	25.93	33.71
PSNR: patient 19	24.61	30.19	31.88
PSNR: patient 20	31.96	33.36	35.87

**Table 10:** Comparison of SSIM for the proposed method with OMS and BS methods for 20 patients

	OMS method	BS method	Proposed method
SSIM: patient 1	0.89	0.91	0.95
SSIM: patient 2	0.76	0.80	0.86
SSIM: patient 3	0.94	0.95	0.98
SSIM: patient 4	0.69	0.74	0.78
SSIM: patient 5	0.83	0.86	0.91
SSIM: patient 6	0.79	0.80	0.89
SSIM: patient 7	0.88	0.92	0.99
SSIM: patient 8	0.75	0.79	0.81
SSIM: patient 9	0.78	0.83	0.84
SSIM: patient 10	0.81	0.82	0.90
SSIM: patient 11	0.92	0.94	0.97
SSIM: patient 12	0.75	0.79	0.83
SSIM: patient 13	0.69	0.70	0.78
SSIM: patient 14	0.77	0.81	0.85
SSIM: patient 15	0.91	0.94	0.97
SSIM: patient 16	0.78	0.79	0.85
SSIM: patient 17	0.86	0.88	0.93
SSIM: patient 18	0.65	0.73	0.77
SSIM: patient 19	0.48	0.53	0.69
SSIM: patient 20	0.87	0.91	0.95

**Table 11:** Comparison of execution time for the proposed method with OMS and BS methods for 20 patients

	OMS method	BS method	Proposed method
Execution time (s): patient 1	76	74	68
Execution time (s): patient 2	54	50	45
Execution time (s): patient 3	36	34	28
Execution time (s): patient 4	52	49	39
Execution time (s): patient 5	31	28	18
Execution time (s): patient 6	49	45	34
Execution time (s): patient 7	55	50	42
Execution time (s): patient 8	81	78	69
Execution time (s): patient 9	42	39	27
Execution time (s): patient 10	35	30	21
Execution time (s): patient 11	59	55	47
Execution time (s): patient 12	66	62	57
Execution time (s): patient 13	54	46	39
Execution time (s): patient 14	44	40	32
Execution time (s): patient 15	63	59	49
Execution time (s): patient 16	23	19	10
Execution time (s): patient 17	62	54	48
Execution time (s): patient 18	41	35	29
Execution time (s): patient 19	57	55	50
Execution time (s): patient 20	43	39	35

**Table 12:** Comparison of SDCT for the proposed method with OMS and BS methods for 20 patients

	SDCT between the proposed method and the OMS technique (s)	SDCT between the proposed method and the BS technique (s)
Patient 1	8	6
Patient 2	9	5
Patient 3	8	6
Patient 4	13	10
Patient 5	13	10
Patient 6	15	11
Patient 7	13	8
Patient 8	12	9
Patient 9	15	12
Patient 10	14	9
Patient 11	12	8
Patient 12	9	5
Patient 13	15	7
Patient 14	12	8
Patient 15	14	10
Patient 16	13	9
Patient 17	14	6
Patient 18	12	6
Patient 19	7	5
Patient 20	8	4

## Discussion

According to the experimental results presented in “Soft biological phantom designed for elastography” and “In-vivo breast images” sections, we can analyze the breast tissue displacement estimation improvement based on the quantitative indicators. The proposed method is applied first to ultrasound B-mode images of two phantoms (that whose stiffness characteristics are the same texture of breast tissue). Secondly, it is applied to ultrasound B-mode images of breast organ of 20 patients with malignant tumor. We note that all results have been verified and validated by two radiologists.

### Soft biological phantoms

Results obtained on simulated data of Soft biological phantoms show that our proposed method retrieves speckle denoising in displacement estimation better than the OMS and BS technique.

The method that has been used to denoise the speckle does not only have an effect on images filtering, but it also has an effect on the quality of the displacement estimation, it has improved the clinical diagnosis of displacement estimation of hard and soft areas.

Compared with our proposed method, the OMS technique appears very noisy with many artifacts that destroy the image quality and prevent the good diagnosis.

The BS technique has more artifacts in displacement estimation tissue and the lesion in phantoms is covered with a large granular of speckle. However, our proposed method can ensure a good speckle suppression, displacement feature preservation and efficiency.

By coupling the shrinkage wavelets and guided filter with monogenic signal, we can not only obtain a strong removing ability of noise, but also we can preserve the details of the displacement estimation tissue without strain degradation. From the index values in Tables 1, 2, 3, 4, 5 and 6, further prove the above description.

It is clear from Table 1, that the SD in pixels of proposed method is lower than OMS and BS methods. This is explained by our solid algorithm to produce a denoising sub-pixel estimation using filtering and monogenic extracted features (orientation, frequency and phase difference).

Table 2 demonstrates that the CNR of our proposed method were significantly higher than those in the OMS and BS techniques. This is explained by the effectiveness of our method to reduce the worm noise artifacts in displacement estimation by using shrinkage wavelets and guided filter, contrary to OMS method that uses a Difference of Gaussian Filter (DoG), or to BS technique that uses a Difference Of Poisson Filter (DoP), and they do not succeed to eliminate the noise affecting the quality of the displacement estimation.

Table 3 shows that the proposed method is the best technique of displacement estimation, which could be used for ultrasound elastography; it gives the highest PSNR value compared with OMS and BS methods. It can be seen that the proposed method gets better results both in terms of speckle reduction and signal detail preservation. This is explained by the implementation of the shrinkage wavelet combined with guided filter instead of the DoG or DoP filters corresponding respectively to OMS and BS techniques.

According to Table 4, the OMS method gets the lowest SSIM value, which means that the output image has less similar structure. The main reason for which is that the DoG filter cannot filter enough noise.

The BS technique has a higher SSIM values than that OMS method, and it do not perform well on similarity. The reason is associated with the DoP filter in BS technique, which in turn failed also to filter the noise.

The proposed method has higher SSIM values than those OMS and BS methods, and we obtained favorable results. It can be readily observed that the coupling between shrinkage wavelets and guided filter combined with monogenic features, outperform other techniques with higher SSIM values in images. The proposed approach has more similar structure and performs better on maintaining the structure of tissue displacement estimation image.

The processing speeds of each method are also studied. The three methods (Proposed, OMS and BS methods) are performed on a Pentium 4, 4.2 GHz with 6 GB RAM using MATLAB.

From Table 5, the running time of our proposed approach is shorter than the running time of OMS and BS methods. Table 6 shows the SDCT between the proposed method and the OMS technique and the SDCT between the proposed method and the BS technique, in order to confirm that our approach is the fastest speed compared to the other methods.

The OMS method takes a lot of time, although the DoG filtering algorithm gives it a more computational time to eliminate artifacts and noise before application of monogenic model. The BS method also requires much more time; the implementation of the DoP algorithm takes more time to filter images before extraction of tissue displacements information. These limits have been exceeded when we have introduced our proposed method. By parallel implementation algorithm of shrinkage wavelets and guided filter in the proposed approach, we can gain a very short time computation.

The computing time to perform displacement estimation depends also on the size of the ultrasound B-mode images (pre and post compression). It is very clear to note that the proposed method improves the estimation of the deformation in ultrasound elastography.

### Breast clinical ultrasound image

To validate quantitatively and qualitatively the effectiveness of our proposed algorithm, we simulated it on ultrasound images of the breast, and we compared it to both methods (OMS and BS methods).

The tissue displacement estimation obtained from cited methods are illustrated in Figs. 3, 4, 5, 6, 7, 8, 9, 10, 11, 12, 13, 14, 15, 16, 17, 18, 19, 20, 21 and 22, the proposed approach gave a better estimate of breast tissue displacement than OMS and BS techniques; the displacement estimation tissue in the case of our developed approach is devoid of artifacts and noise with good preservation of breast texture tissue. We notice a good accuracy of tumor location, the lesion is well defined in its position, no noise, no artifact, and the image is clear enough to be read by the doctor to ensure a more accurate diagnosis.

For a quantitative assessment of results, the SD, CNR, PSNR, SSIM, execution time and the SDCT are computed for each method.

It is seen from Table 7 that the SD in pixels of proposed method is lower than that obtained with OMS and BS methods. Thanks to the use of shrinkage wavelets and guided filter algorithm which eliminates all notions of noise in the image, coupled with the monogenic model which estimates a sub-pixel displacement with good preservation of texture. This explains the smaller value of SD in the proposed model. Our strategy has announced its success from these results, nor the OMS method that used a DoG filter can assume a good sub pixel precision estimation in front of our proposed method, neither can the BS method do it perfectly.

Since the noise is much diffused in the image, the OMS and BS methods have not succeeded in eliminating perfectly the noise. So in all the cases, the error will be very great, which explains these obtained results.

A further fundamental point concerns CNR of proposed method, OMS and BS methods, is calculated, a criterion often involved in the evaluation of ultrasound image processing techniques [22, 23].

Table 8 demonstrates that the displacement estimation with proposed model gives a higher CNR than these B-mode images, OMS and BS techniques.

Despite the fact that the CNR is good in both methods: OMS and BS techniques, the CNR is better in the case of the proposed model, better contrast, better filtering, good visibility of the image details.

These results are explained by the fact that the noise is eliminated by two steps in the developed model; the first step is related to the shrinkage wavelet which eliminates the noise according to the adapted threshold, and the second step consists of filtering the noise still existing in the low frequencies, information still containing noise. In the other methods (OMS and BS) we find only the DoG and the DoP filters that processes the images in a single band, band-pass filtering, which does not solve perfectly the noise problem.

Our proposed strategy will solve much more the problems of artifacts and noise, before integrating monogenic model of displacements estimation and that will necessarily improve the results in ultrasound elastography.

From Table 9, it can be seen that the OMS method obtains the lowest PSNR value. The reason for which, is that it does not filter the large noise in the low frequency band. The BS technique also does not perform well according to PSNR. This is explained by the DoP filter deficiency in the non-preservation of the details in the low frequency component with imperfect filtering of noises.

For the proposed method; it provides a better PSNR value than those in the OMS and BS techniques and it demonstrate a strong de-speckling ability. The filtering technique adopted by the selection of the wavelet threshold and the guided filter in the proposed approach, contributes effectively to preserve the details in all frequency bands, which improves the resolution of displacement estimation image, and perfectly reduces the noise.

It can be observed from Table 10, that the proposed method outperforms other techniques with the highest SSIM value. The BS technique also provides a satisfactory result, but still having lower SSIM value than our proposed method. However, the reason why OMS technique method did not get a favorable SSIM value, is that the DoG filter may suffer from speckle noise in the low frequency band, as often appears on an edge. The proposed filtering strategy is suitable for removing the speckle in ultrasound images and improving the image qualities as well, which explain the satisfactory result obtained with proposed approach.

Another evaluation criteria using computational time is calculated for these three methods (Proposed, OMS and BS methods) to demonstrate the rapidity of our method. The simulation results were done on a MATLAB executed on a desktop PC with a Pentium 4, 4.2 GHz, 6 GB RAM running Windows 7 using MATLAB.

It is seen from Tables 11 and 12, that the proposed model is much faster speed than those OMS and BS methods. The OMS technique suffers from a wide range of noise covering B-mode images, this noise will require much processing steps by the filter to denoise it, and it will necessarily take a longer computation time to get the displacement estimation result.

The weak point of the BS method is in its complex algorithm, in terms of non-perfect filtering and very long calculation time to estimate the tissues displacements, so the BS method cannot meet the requirements of ultrasound elastography and it takes a lot of time running.

The parallel implementation of our proposed algorithm via non-iterative fast filtering accelerates the displacement estimation result.

Given all this, it is very clear to confirm the excellence of the proposed method as a quick method in the case of displacement estimation of breast tissue that is suitable to be used in ultrasound elastography.

In this article we have improved the OMS technique used in quasi-static ultrasound elastography, we have adapted the use of shrinkage wavelet and guided filter with monogenic model to obtain a good qualitative and quantitative results.

We have succeeded in improving breast tissue displacement; our proposed model will solve several problems related to noise and low contrast in ultrasound elastography images. The implementation of our method will help doctors for a reliable and accurate diagnosis for the evaluation of tumor stiffness with more comfort due to its rapid execution time.

## Conclusion

In this paper, we proposed a new method for the analysis of breast tissue displacements estimation, the adopted methodology has improved the OMS technique used to estimate the strain in Breast ultrasound elastography. The new utility of proposed framework leads to improve the diagnosis of breast pathology.

A shrinkage wavelets and guided filter combined with monogenic signal technique between a pair of images is presented, to estimate with accuracy the breast tissues deformation.

The proposed approach was found very effective in tissue displacements estimation in ultrasound elastography. It can preserve correct texture details and reduce artifacts generated by speckle noises and target movement.

Moreover, the implementation of proposed approach is discussed, using synthetic ultrasound elastography Phantom and in vivo B-mode breast images of 20 patients with malignant tumors. It has been observed that the proposed method works much better compared to OMS and BS techniques and gives better SD, higher CNR, greater PSNR, more suitable SSIM and shorter run time than OMS and BS methods. Therefore, our selected method gives encouraging results and may facilitate the breast tumors diagnosis.

Generally, the proposed enhancement method is valuable in improving the quality of displacement estimation, used in breast ultrasound elastography, for better visualization and clinical assessment.

## Abbreviations

OMS: old monogenic signal
BS: B-spline
SD: standard deviation
CNR: contrast to noise ratio
PSNR: peak signal to noise ratio
SSIM: structural similarity
DoG: Difference of Gaussian Filter
DoP: Difference of Poisson Filter
MSE: mean squared error
SDCT: standard deviation of computational time
